# Identification of Metabolic Engineering Targets through Analysis of Optimal and Sub-Optimal Routes

**DOI:** 10.1371/journal.pone.0061648

**Published:** 2013-04-23

**Authors:** Zita I. T. A. Soons, Eugénio C. Ferreira, Kiran R. Patil, Isabel Rocha

**Affiliations:** 1 Institute for Biotechnology and Bioengineering, University of Minho, Braga, Portugal; 2 Structural and Computational Biology Unit, EMBL-Heidelberg, Heidelberg, Germany; Virginia Commonwealth University, United States of America

## Abstract

Identification of optimal genetic manipulation strategies for redirecting substrate uptake towards a desired product is a challenging task owing to the complexity of metabolic networks, esp. in terms of large number of routes leading to the desired product. Algorithms that can exploit the whole range of optimal and suboptimal routes for product formation while respecting the biological objective of the cell are therefore much needed. Towards addressing this need, we here introduce the notion of structural flux, which is derived from the enumeration of all pathways in the metabolic network in question and accounts for the contribution towards a given biological objective function. We show that the theoretically estimated structural fluxes are good predictors of experimentally measured intra-cellular fluxes in two model organisms, namely, *Escherichia coli* and *Saccharomyces cerevisiae*. For a small number of fluxes for which the predictions were poor, the corresponding enzyme-coding transcripts were also found to be distinctly regulated, showing the ability of structural fluxes in capturing the underlying regulatory principles. Exploiting the observed correspondence between *in vivo* fluxes and structural fluxes, we propose an *in silico* metabolic engineering approach, iStruF, which enables the identification of gene deletion strategies that couple the cellular biological objective with the product flux while considering optimal as well as sub-optimal routes and their efficiency.

## Introduction

Microorganisms that produce a desirable product, either naturally, or because they have been engineered through insertion of heterologous pathways, often have low yields and productivities. Only after the introduction of appropriate genetic modifications, production strains may become available that can meet the demands of economic production [1], [2]. Availability of genome-wide information on cellular metabolic networks has opened the possibility of *in silico* analysis for identifying the required genetic engineering strategies towards increased productivity, an approach often termed ‘*in silico* metabolic engineering’. However, given the complexity of metabolic networks in terms of their structure and regulation, identification of optimal strategies for redirecting fluxes towards desired products is a challenging task.

Several solutions to the *in silico* metabolic engineering problem have been proposed in recent years. The OptKnock algorithm [3] represents one of the first model-based frameworks for suggesting gene knockouts leading to the overproduction of a desired metabolite. By using an elegant bi-level optimization strategy, OptKnock searches for an optimal set of gene (reaction) deletions that maximize the flux towards a desired product (Design Objective), while the internal flux distribution is still operated such that the growth (or another linear Biological Objective) is optimized, which in turn is simulated by using Flux Balance Analysis (FBA). Algorithms such as OptGene [4] [5] further expand this approach and allow for the use of relevant non-linear design and/or biological objective functions, such as MoMA [6]. In essence, the basic idea behind these algorithms is to couple the desired design objective function with the biological objective function inherent to the system. Practical relevance of the algorithms based on this idea is becoming apparent through experimental verification of the predictions, including overproduction of lycopene [7], vanillin [8], and sesquiterpene [9]).

One of the key requirements for successful metabolic engineering target identification is the ability to predict biologically meaningful flux distributions following genetic perturbations such as gene knockouts. In OptKnock/OptGene, this requirement is explicit in terms of the biological objective function included in the optimization problem. Current approaches typically assume that microbes have evolved for achieving a flux distribution that leads to maximum growth (or another flux-based objective). The biological feasibility of a solution thus depends on the validity of the assumption that the formulated objective function correctly represents the system. Although the assumption of optimality for a wild-type microorganism is justifiable, it may not be valid for mutants [6], [10]. Therefore, it is often observed that the engineered cells do not function according to the predicted optimal pathway. In such cases, either an alternate optimal solution may be biologically more meaningful than the predicted distribution, or several of the available routes, including optimal & sub-optimal, are simultaneously utilized *in vivo*. Additionally, the presence of futile cycles can cause certain fluxes to have an infinite range of variation [11], being hence difficult to estimate.

Linear programming based methods can be used to tackle some of the above-mentioned limitations, for example, by using flux-cone sampling methods [12] or by calculating the lower limit on the design objective function [4] [13]. Another attractive approach to this end is the use of pathway analysis methods, such as elementary modes [14]. Pathway analysis has the advantage of identifying all pathways inherent to a metabolic network and thus determining alternate flux distributions with equivalent yields. The availability of methods that tackle the *in silico* metabolic engineering problem using pathway analysis is limited to few. Trinh and co-workers [15] proposed sequential deletion of reactions to enforce a desired elementary mode, while Melzer et al. [16] computed targets by correlating the desired flux with the flux through the intracellular reactions of the elementary modes matrix. Boghigian et al. [17] used a genetic algorithm to find gene deletions under the assumption of minimization of Gibbs energy of the macroscopic pathways. Hädicke and Klamt [18] and Bohl et al. [19] proposed an interesting approach, termed “CASOP”, to enhance productivity while producing biomass by sequential deletion or over-expression of reactions. Additionally, Hädicke and Klamt [20] proposed a gene deletion strategy based on minimal cut sets to identify a minimal set of knockouts disabling the operation of a specified set of target elementary modes, while keeping a set of desired modes.

In this study, we aim at combining the advantages of both objective function-centered and pathway enumeration-centered approaches. To this end, we first address the question of biological relevance of sub-optimal routes and flux distributions predicted by computational methods. We introduce the notion of structural fluxes, which account for a biological objective function and are derived from the enumeration of all pathways in a given metabolic network. Structural fluxes are inspired from the concept of control effective flux (CEF) that uses efficiency and elementary modes to understand changes in transcriptional regulation [21], [22] and has been modified to estimate flux changes [23] for growth on different substrates.

We show that structural fluxes are good predictors of experimentally measured fluxes in *Escherichia coli* and *Saccharomyces cerevisiae*. Building upon the ability of structural fluxes to predict genetically perturbed biological networks, we propose an *in silico* metabolic engineering algorithm, iStruF, where the objective is to identify deletion targets that increase the structural flux of a desired product. iStruF leads to solutions that couple biological objectives, such as growth, with product formation while considering optimal as well as sub-optimal routes and their efficiency. As a biotechnologically relevant case study, we present the results of iStruF for improving production of ethanol and succinate in baker’s yeast. Finally, we discuss the use of Generating Vectors (GVs) [24] instead of elementary modes (EMs) for the calculation of structural fluxes towards enabling the application of iStruF to large-scale metabolic networks.

## Methods

**Figure 1:**
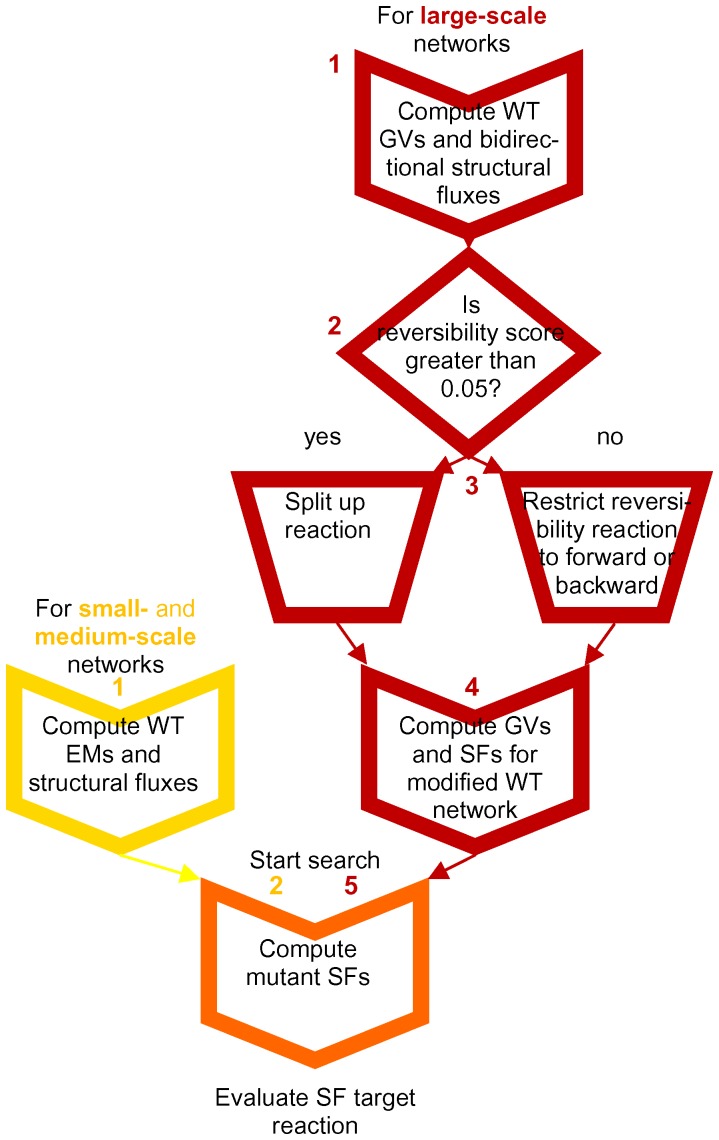
Procedure for finding targets of reaction deletions based on structural fluxes. The structural fluxes are computed from elementary modes (EMs, yellow) or from generating vectors (GVs, red) to facilitate larger-scale application. The yellow and red boxes are performed once to compute a biologically relevant set of structural fluxes for the wild-type (WT) network. The orange box presents the iterative computation of the structural fluxes for mutants with one or multiple reaction knockouts through re-computing the StruFs from the mode efficiencies that do not contain the deleted reaction(s) using Eqs. (3–5) without re-computing the EMs or GVs.

An overview of the proposed *in silico* metabolic engineering procedure is given in Fig. 1. It involves computation of elementary modes (for small-scale networks, yellow box) or GVs (for larger models, red boxes in Fig. 1), and, for this last case, a methodology to deal with reversible reactions, followed by the computation of the structural fluxes. The metabolic engineering algorithm (orange box) involves evaluation of different knockout mutants to find the best one (the one that maximizes the structural flux of the desired product).

### Control Effective Fluxes

In the original formulation of CEF [21], the efficiency *ε* of each elementary mode *i* is defined as the ratio of the EM’s output *e* (the cellular objective, in many cases growth *µ* and/or ATP production) to the investment required to establish the EM (the sum of the absolute flux values in the EM) for a specific carbon source:
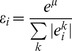
(1)


The control effective flux of each reaction *k* is then obtained by the weighted average of the product of mode-specific efficiencies and reaction-specific fluxes over the sum of all mode efficiencies:
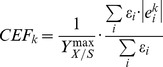
(2)where 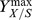 denotes the maximum yield obtained for the specified cellular objective (biomass (*X*) production and/or ATP generation for cellular maintenance on substrate *S*). In order to find a measure that can predict fluxes across mutants, we propose three modifications to the definition of CEFs and thereby introduce the concept of Structural Fluxes.

### Structural Fluxes

Two flavors of structural flux definitions are introduced, namely, SF and StruF. SF is introduced in the context of application on a single given network, while StruF is introduced for cases where comparison across different networks (e.g. wild-type versus mutant) is required.

#### Structural fluxes and reaction reversibilities

In metabolic systems, the cellular network of reactions, together with constraints on the reversibility of enzymes, determine the space of all possible steady-state phenotypes. In actuality, the cell does not invoke the large majority of those in a given condition. For example, for growth on a given substrate, several of the reversible reactions across the network are usually constrained to either backward or forward directions. We use this fact in order to derive heuristics for restricting reaction directionalities. Such restrictions, together with splitting of bi-directional reactions into two unidirectional reactions allowed us to obtain a pointed cone and thereby to avoid re-computation of GVs following each network perturbation (gene deletions).

In the concept of structural fluxes, we first split up the fluxes of reversible reactions into forward (f) and backward (b) directions as a way to consider biologically relevant directionalities for growth on a given substrate:
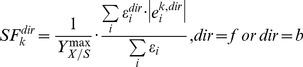
(3)


Our hypothesis is that the greatest SF of a particular reaction (either forward or backward) matches the directionality *in vivo*. Hereto, we define the reversibility score *rs* of a reaction as the smallest divided by the greatest SF:
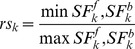
(4)


A reversibility score of 0 indicates that the reaction is irreversible and a score of 1 indicates that the reaction may be active in both directions. We assume that the directionality of a particular reaction may flip in mutants as compared with wild-type for a high reversibility score; otherwise we assume the directionality to be equal for mutant and wild-type (irreversible). Based on the reversibility score (Eq. 4), we impose additional restrictions on the reaction directionalities (on top of the directionalities reported in the models): split up the reaction above a threshold of 0.05; otherwise restrict the reaction to either forward or backward direction.

#### Normalization for StruFs

Although elementary modes are normalized to the substrate uptake rate, control effective fluxes are not. SF and CEF values tend to be larger for smaller networks. If the CEFs as such would be used to find deletion targets for the production of a target metabolite, the algorithm would tend to minimize network size, besides maximizing product formation. It would thereby favor deletion of reactions that are present in many elementary modes and lead to a biased set of deletion targets. We show the importance of normalization in a case study on succinate production using yeast in Table S3 of Supplement S4. Hence, the absolute CEF values are not comparable across networks [22] and an appropriate normalization is necessary. When benchmarked against the flux dataset from Ishii et al. [25], the best normalization to predict the fluxes in *E. coli* was found to be the maintenance reaction (i.e. ATP requirement for maintenance). However, this normalization is not suitable for the identification of metabolic engineering targets, as the algorithm would tend to minimize the use of the maintenance reaction in the mutant EMs when maximizing the product formation. We chose the glucose (substrate) uptake rate as the normalization factor for predicting fluxes and in metabolic engineering applications:
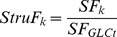
(5)


Zhao and Kurata [26] also introduced a methodology that relies on modifications of control effective fluxes, though the application to multiple knockouts is not apparent, as these predicted fluxes cannot be compared across networks without appropriate normalization.

### Datasets and Model Alignment

Metabolic networks of two different species are used in this work, a model of *E. coli* central carbon metabolism (Supplement S1) and another of *S. cerevisiae* central carbon and amino acids metabolism [22].

To compare the measured and predicted reaction directionalities, we selected a dataset that represents growth on different substrates as the sole carbon sources for *S. cerevisiae* [27], since directionalities of some reactions flip depending on the used substrate, e.g. from glycolysis to gluconeogenesis.

With respect to the flux data for gene deletion mutants, Blank et al. [28] represents the largest such dataset for yeast, covering 36 single gene deletions plus the reference strain, grown on glucose in batch fermentations. In case of *E. coli*, we use the data from Ishii et al. [25] that reports flux measurements for 24 single gene deletions and the wild-type during growth on glucose in continuous cultures at a dilution rate of 0.2 *h^−1^*. For both datasets, we only considered data for a subset of gene knockout mutants, only corresponding to genes for which no isozymes exist. This filtering is necessary as the individual contributions by each of the isozymes are not distinguishable from the experimental data, as well as in the current computational models.

The models used by Blank et al. [28] and Ishii et al. [25] for *in vivo* flux estimations are much smaller and simplified versions of the ones that we used for StruF calculations. Differences are due to compartments, isozymes and lumped reactions. Details on the procedure used for the alignment of reactions between the dataset models and StruF models are given in Tables S1 and S2 in Supplement S2. Overall, it was possible to align 32 reactions in the *E. coli* model and 29 in the *S. cerevisiae* model. Alignment of the gene-reaction relations is also given in Supplement S2.

### Implementation

All calculations were performed using Matlab 7.1 (Mathworks Inc.). GVs were computed using METATOOL [29] and the EMs using efmtool [30]. The ROBPCA function from the Matlab toolbox LIBRA [31] was used to classify outliers in gene expression pattern. Matlab implementation of iStruF and structural flux calculation is available upon request.

## Results

### Structural Fluxes

In this section, we compare the calculated structural fluxes with the experimental data from *in vivo* ^13^C labeling experiments for *S. cerevisiae* and *E. coli*: both with respect to reversibility of reactions for growth on different substrates, and with respect to the measured fluxes across different mutants.

#### SFs and reaction reversibilities

We evaluate reaction directions in the central carbon metabolism of *S. cerevisiae* under three different conditions: growth on glucose, glycerol, or acetate as the sole carbon sources. Under these conditions, the net directionality of some of the fluxes varies. For instance, growth on glucose involves a net flux through the glycolysis from DHAP to PEP, whereas growth on acetate involves the reverse (net flux through the gluconeogenesis). As the same enzymes are used in all cases, the favored flux directions estimated based on the structural flux reversibility scores should also change.

**Figure 2:**
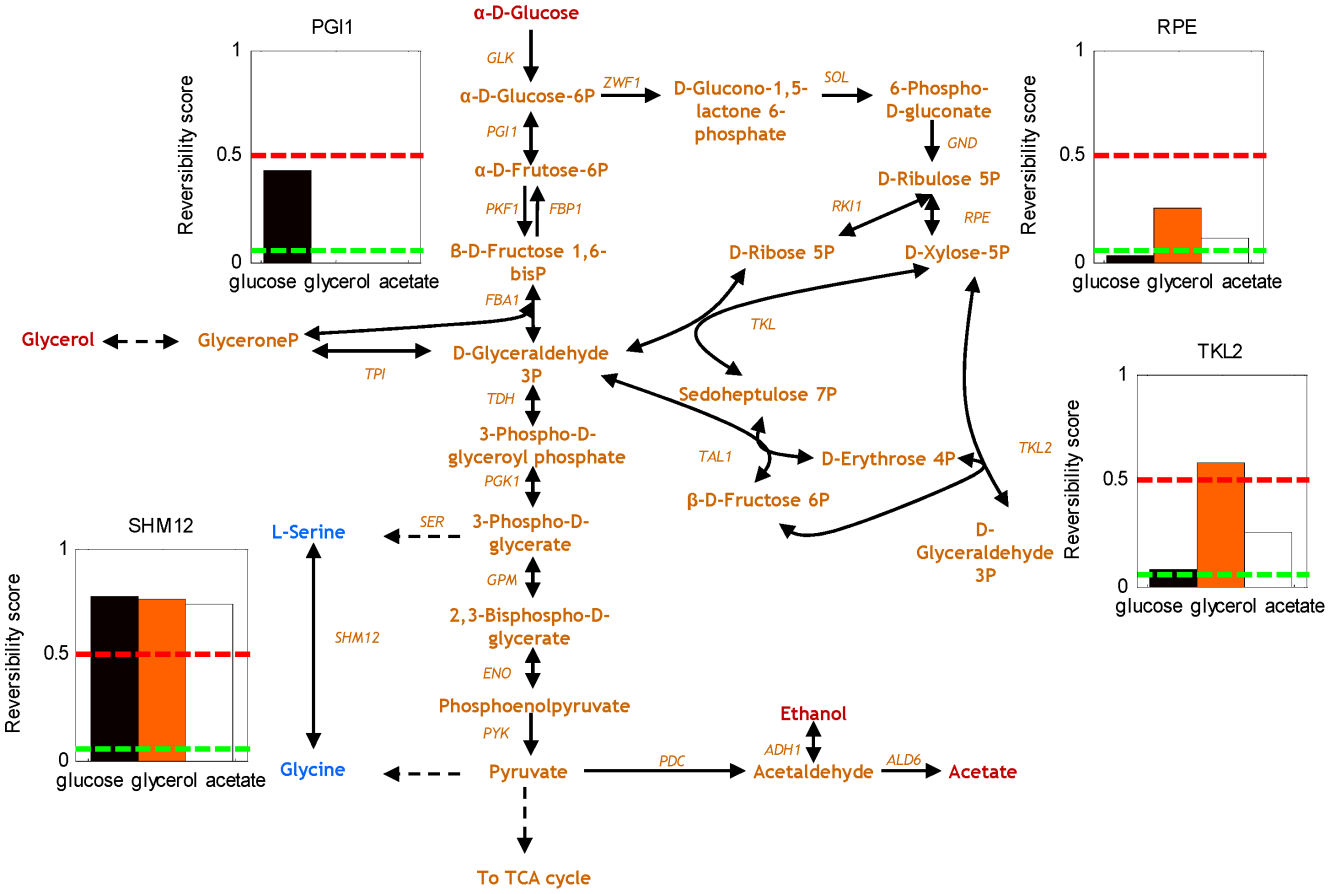
Metabolic map of wild-type *S.* *cerevisiae*. Central carbon metabolism (orange), amino acids metabolism (blue), and extracellular metabolites (red). The graphs show the predicted reversibility scores using generating vectors for four out of 26 potentially reversible reactions for growth on glucose, glycerol, and acetate. A reversibility score of 0 indicates that the reaction is irreversible and 1 that the reaction may be active in both directions with equal flux on a particular substrate. The red line indicates the level of 0.5, below which all reaction directionalities are correctly predicted. The green line indicates the threshold of 0.05, above which a reversible reaction is split up into a forward and backward reaction in the approach based on generating vectors.

We observed that the direction given by the greatest structural flux when the reversibility score is smaller than 0.5 matches well with the measured reaction directionality for growth on glucose (100% match), glycerol (90% match), and acetate (100% match) as sole carbon sources. SF thus correctly captures the experimental observations on the reaction directionalities. Figure 2 shows the reversibility scores for reactions in the yeast model that display large changes in the score across different conditions. The net flux for most reactions, as predicted by their structural flux values, were in accordance with the measured data of Zhang et al. [27]. With respect to growth on glucose, only eight reactions, out of 26 potentially reversible reactions, can carry a net flux in both directions; moreover, for four reactions a very small flux can exist. Thus, most of the potentially reversible reactions can be considered irreversible for growth on a particular substrate.

An alternative way to constrain reaction reversibility and the number of feasible elementary modes can be obtained by assigning reaction directionalities on thermodynamic grounds. To compare reversibility scores to thermodynamics-based analysis, we used a network thermodynamics approach [32] [33] to restrict the reaction reversibilities and subsequently remove infeasible pathways. Supplement S7 presents the methods and discusses the results. Depending on the available metabolite measurements and their uncertainties in the different datasets, the directionalities of particular reactions are restricted and a reduction of up to 57% in the number of computed pathways was obtained for an *E. coli* model (Fig. S5 and table S7 in Supplement S7).

Assignment of reaction directionalities based on reversibility scores, on the other hand, puts constraints on all but four highly reversible reactions in *E. coli,* thus allowing for a much smaller set of relevant pathways. In addition, thermodynamics analysis provides constraints on reaction reversibilities in particular conditions, whereas the extrapolation of these restrictions to mutant strains is still an open question. Reversibility scores, on the other hand, give a measure for potential variability of the fluxes in the opposite direction. Furthermore, reversibility score calculations do not require measurements of intracellular metabolite concentrations, which are often lacking or incomplete in many practical cases.

#### Biological objectives

**Figure 3:**
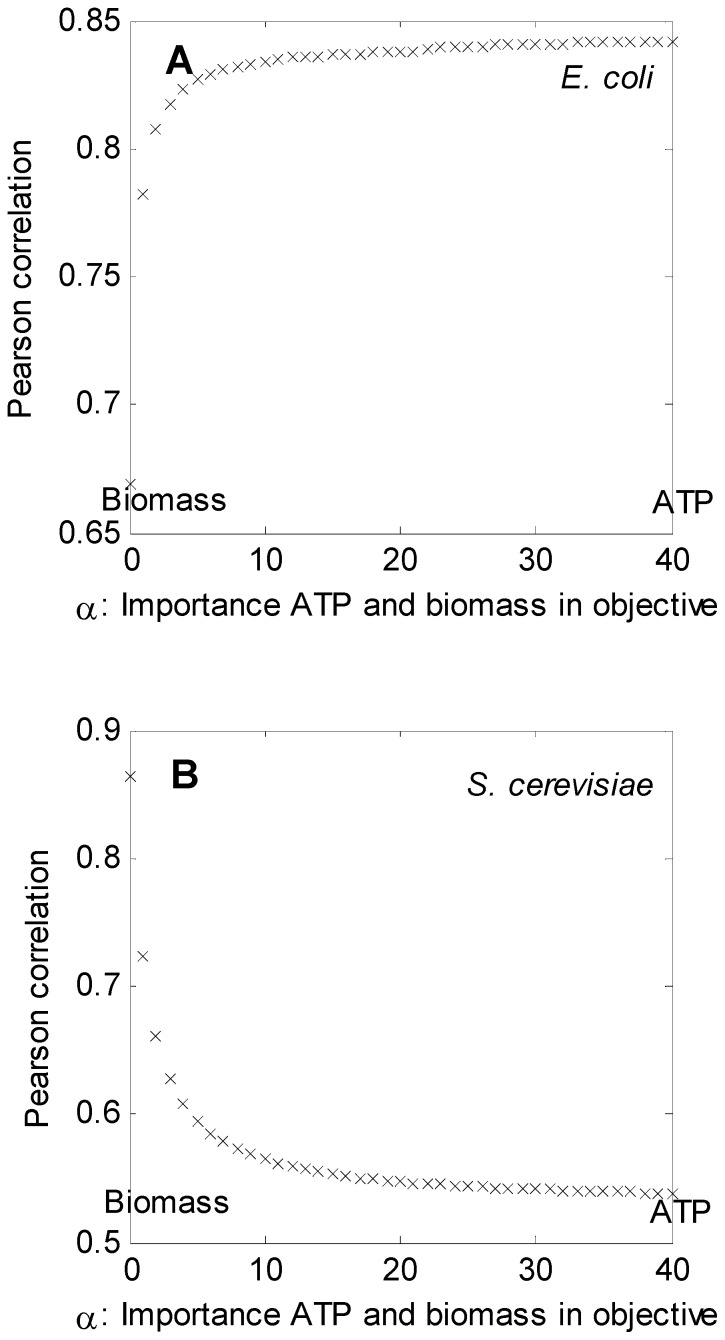
Biological objectives. Average Pearson correlation coefficient of the predicted structural fluxes versus measured ^13^C fluxes for different degrees of importance of biomass and ATP in the objective. **A.** for *E. coli* mutants **B.** for *S. cerevisiae* mutants.

Metabolic networks in living cells can function according to various different biological objectives depending on the organism in question and its genetic and environmental context. Although so far, biological objectives have been elucidated only for a few organisms, in the perspective of microbial metabolic engineering, it is desirable to couple formation of the desired product to growth (i.e. biomass formation). Hence, the biological objective used for structural fluxes calculation must at least contain growth. In the current study, we use growth as the objective for *S. cerevisiae* [34] and growth and ATP generation for *E. coli* [10]. For *E. coli*, we weighted ATP 20 times more than biomass, since ATP production was found to explain intra-cellular fluxes in several *E. coli* mutants [10]. The presented results are robust regarding the precise choice of this weighting. We independently confirmed the goodness of the choice of these objectives by assigning different degrees of importance to biomass and ATP in the cellular objective and comparing the predictions with measured ^13^C fluxes (Fig. 3 and Supplement S3). We found that the Pearson correlation coefficients between the predicted and measured fluxes were almost optimal for the proposed objectives.

#### StruFs and generating vectors

**Figure 4:**
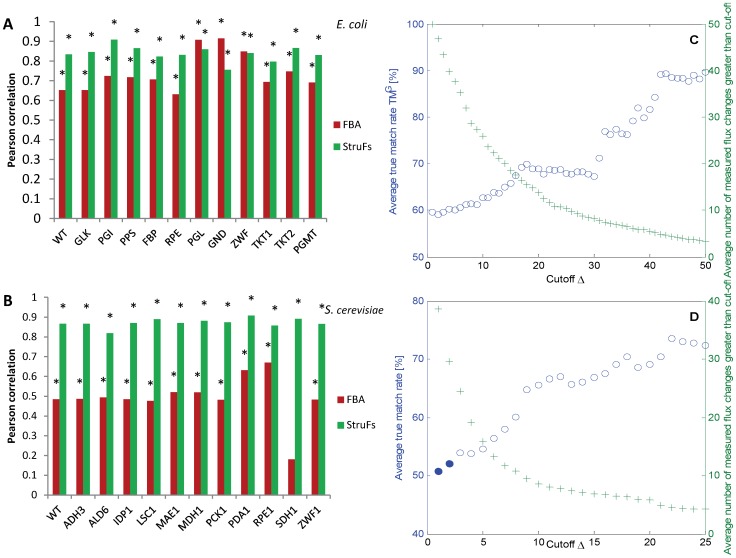
Structural fluxes based on elementary modes. **A.–B**. StruFs compared with FBA for the *E. coli* and *S. cerevisiae* mutants, respectively. The asterix * indicates a significant correlation between predictions and measurements. **C.–D.** StruFs across mutants. Right axis (+): Average number of measured flux changes across all mutants greater than cut-off (green); Left axis (o): Average true match rate TM^G^ (Eq. 6). The predictions that are significant compared with random are indicated with open symbols (o), the non significant predictions with closed symbols (•).

A limitation of the use of the CEF and StruF definitions based on EMs is current computational intractability of EMs for large-scale metabolic networks [35]. This problem can be tackled to some extent by sampling the elementary modes ([19], [36], [37]. In this work, we propose the use of the minimal generating set (Generating Vectors, GVs), which are a subset of EMs and allow enumeration in polynomial time [24]. Details on the use of GVs to compute structural fluxes for metabolic engineering purposes, along with an empirical validation that structural fluxes based on generating vectors are good predictors of intracellular fluxes in mutants, can be found in Supplement S5. The solutions were also compared with and found superior to FBA in terms of higher correlation with experimental data (Figs. 4AB, Tables S4–S5, and Figs. S1A and S1B in Supplement S5). In addition, phenotypic phase plane analysis showed that the yields of generating vectors have similar distribution as those of elementary modes (Figs. S2–S3 in Supplement S5). We also verified that reaction participation in the modes is highly correlated for generating vectors and elementary modes (Fig. S4 in Supplement S5). These results strongly suggest that generating vectors are representative of the full set of EMs for the use in the optimization of metabolic networks.

#### StruFs reflect *in vivo* flux measurements across mutants

Many knockouts in the datasets obtained from literature represent reactions catalyzed by isozymes or that carry a small flux in the wild type (less than 10% of the glucose uptake rate). Consequently, for the purpose of comparing fluxes of particular reactions across different mutants, variability in the data is a limiting factor. With respect to our main objective of identifying metabolic intervention strategies, however, it is more important to determine whether a given flux will be increased or decreased in comparison with the wild type or with another mutant, as opposed to flux magnitude comparisons within the same mutant. We therefore evaluate the performance of the predictions of the structural fluxes in comparison with the ^13^C-based *in vivo* flux measurements in the following manner: for every pair of strains, all experimentally measured flux changes greater than a cut-off value *C* (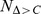) were selected, where the flux values are normalized by the glucose uptake rate. For these selected fluxes, we checked if the measured changes match the up- or down-regulation as predicted by the structural fluxes (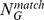) and thereby computed the true match rate as:
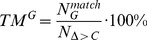
(6)


Since for metabolic engineering purposes it is also of importance to determine whether a given flux remains constant in comparison with the wild type (or with another mutant) we define a similar measure for the unchanged fluxes:
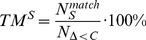
(7)


As expected, the numbers of flux changes greater than the cut-off value decreased with increasing cut-off (Figs. 4CD). Interestingly, the true match rate TM^G^ improved with increasing magnitude of flux change, demonstrating that structural fluxes successfully capture large flux changes in the network. Furthermore, the overall prediction of up or down regulation of fluxes across mutants was also found to be good (Table 1 and Table S6 in Supplement S6). Table 1 also shows that the average true match rate for the unchanged fluxes with a TM^S^ cut-off of 15% is 71% and with a cut-off of 40% is 91% in *E. coli*. Higher true match rates were obtained in yeast: 85% for a cut-off of 15% and 95% for a cut-off of 40%. We expect that further elevated true match rates may be obtained for datasets with more flux variability.

**Table 1:** True match rate of the predicted flux changes (Eq. 6) and of the constant fluxes (Eq. 7) using a cut-off of 15% (and 40% in parenthesis) based on experimental data for *E. coli* using EMs, where 100% represents the glucose flux.

Reaction	True match rate TM^G^ [%]	Number of flux changes greater than cut-off	True match rate TM^S^ [%]	Number of flux changes smaller than cut-off
GLCt	−*	0 (0)	100 (100)	66 (66)
PGI	64 (−*)	14 (0)	59 (67)	41 (55)
PFK-FBP	100 (100)	11 (11)	60 (67)	55 (55)
FBA	82 (82)	11 (11)	33 (67)	55 (55)
TPI	82 (82)	11 (11)	33 (67)	55 (55)
GAPDH	100 (100)	11 (11)	56 (67)	55 (55)
PGM	100 (100)	11 (11)	56 (67)	55 (55)
PYK-PPS	59 (67)	37 (9)	48 (96)	29 (57)
G6PDH	95 (100)	19 (10)	47 (80)	36 (45)
PGDH	63 (56)	19 (9)	47 (63)	36 (46)
RPE	58 (−)	12 (0)	47 (100)	43 (55)
RPI	71 (−)	7 (0)	90 (97)	100 (66)
TK1	71 (−)	7 (0)	90 (100)	59 (66)
TA	71 (−)	7 (0)	90 (100)	59 (66)
TK2	71 (−)	7 (0)	93 (100)	59 (66)
PDH	63 (0)	35 (1)	87 (100)	31 (65)
CS	67 (86)	36 (7)	57 (100)	30 (59)
ACONT	72 (86)	36 (7)	63 (100)	30 (59)
ICDHy	57 (88)	42 (16)	75 (100)	24 (50)
AKGD	57 (82)	42 (17)	75 (100)	24 (49)
SUCD1i-FRD	84 (100)	37 (7)	93 (100)	29 (59)
FUM	76 (100)	37 (7)	66 (100)	29 (59)
MDH	57 (100)	37 (4)	66 (100)	29 (62)
PPC-PPCK	20 (−)	10 (0)	63 (94)	56 (66)
ME1	− (−)	0 (0)	59 (100)	66 (66)
ICL	32 (−)	22 (0)	89 (100)	44 (66)
MALS	32 (−)	22 (0)	89 (100)	44 (66)
PTAr-ACS	− (−)	0 (0)	100 (100)	66 (66)
EDA	25 (−)	4 (0)	50 (6)	60 (10)
LDH	− (−)	0 (0)	100 (66)	100 (66)
ADHE	− (−)	0 (0)	100 (66)	100 (66)
Average	67 (83)	18 (5)	71 (91)	44 (57)

* The hyphen “−” indicates that no measurements were available.

As a performance indicator, we tested the true match rates (TM^G^) from StruFs against random predictions in a binomial test with the expected true match rate for the random model being 50%. Concerning *E. coli*, Fig. 4C shows that the predictions from StruFs are all significant (p-value <0.05) with an average p-value of 4.4E−10. In particular, the predictions for high flux changes are more significant. Concerning *S. cerevisiae*, Fig. 4D shows that the predictions from StruFs are significant for flux changes greater than 2% with an average p-value of 1.9E−2.

Few particular fluxes were found to consistently show a poor true match rate, such as the glyoxylate shunt (reactions ICL and MALS). We hypothesized that these fluxes are likely to be regulated by other means than those taken into account by the structural properties of the network. For example, the transcriptional regulation for these fluxes may display a different pattern than for the other fluxes. To test this hypothesis, we employ statistical analysis to detect enzyme-coding transcripts that are differently regulated by using mRNA measurements from the *E. coli* dataset [25]. In particular, robust principal component analysis [31] was used to classify the genes with expression patterns different than average.

**Figure 5:**
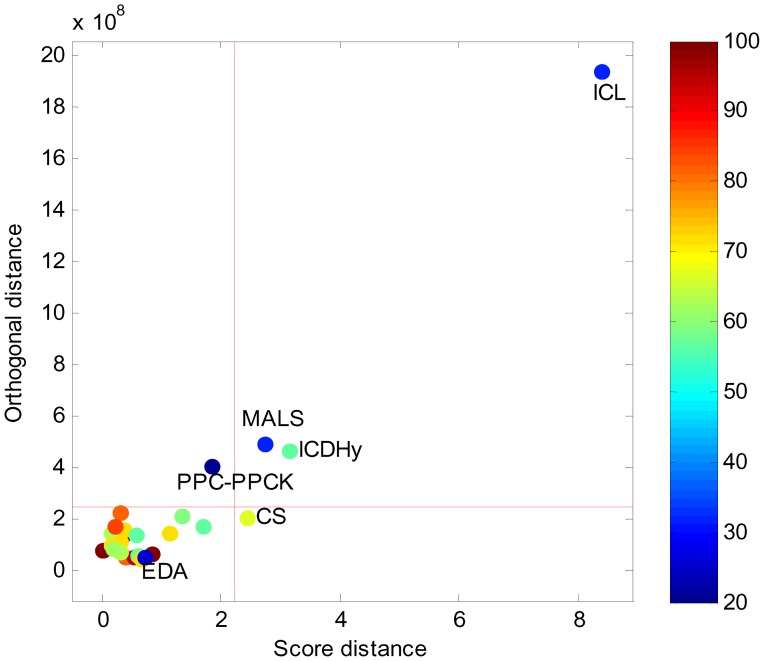
Principal component analysis on the ***E.*** **coli**** **mRNA dataset** [**25**] **.** **** Score outlier map for one principal component (explaining 94% of the data). The observations are colored according to their true match rate TM^G^ (Eq. 7) at a cut-off of 15%. Outliers in the transcripts and bad StruF predictions are labeled.

Outliers of the principal component analysis are marked in Fig. 5, as well as the outliers with the low true match rate from StruF analysis. Most of the regular data points (i.e. with small score distance and orthogonal distance) were predicted well by structural fluxes (yellow-red observations/Table 1). We performed a Wilcoxon rank sum test to test whether the outliers (defined from the principal component analysis) come from a distribution with equal means compared to the non-outliers. The test showed that the mean values are significantly different considering six outliers (ICL, MALS, PTAr, ICDHy CS, and PPC): p = 1.0E−2. The poorly predicted glyoxylate shunt from StruFs corresponds well with the outliers ICL and MALS. PTAr-ACKr is an outlier in transcription profiles, but neither acetate production nor consumption was measured for any of the knockouts under the experimental conditions of low dilution rate and hence does not appear in the figure. The structural flux through the Entner-Dourodoff pathway was also poorly predicted; however, it was not found to be an outlier in the transcript measurements. We here note that only few flux measurements were available for EDD. Adding *a priori* information on regulation, e.g. the absence of fermentation reactions at low specific growth rates, would improve the predictions, because the structural fluxes represent a “capacity” for each flux, whereas the measurements reflect a particular situation.

### *In silico* Metabolic Engineering

The objective of the metabolic engineering algorithm iStruF is to identify deletion targets that increase the structural flux of the desired product. For the metabolic networks under study, it was feasible to perform an exhaustive search and compute all potential reaction deletions up to the combinations of three reaction deletions. For each gene deletion mutant, the structural fluxes of each reaction (Eqs. 3, 5) were recomputed by excluding the modes that contain the deleted reaction(s). For large-scale networks or higher number of gene deletions, search algorithms like controlled random search [38] or genetic algorithms [39] would be needed in order to find the target deletions.

As case studies for the *in silico* metabolic engineering strategies, we selected i) production of ethanol through single reaction deletions and ii) production of succinate through triple deletions in baker’s yeast. Both compounds are interesting bio-based alternatives for replacing products that are currently manufactured from fossil fuels.

#### Ethanol production in yeast

While considering the biomass producing modes, we note that the maximum theoretical structural flux towards ethanol is 1.68 mole ethanol/mole glucose. Whereas, the structural flux for the wild-type is 0.36, thus setting a theoretical limit of 4.5 fold improvement in production (at the expense of a halved growth). We present the results of an exhaustive search for single reaction deletions using iStruF and contrast it with the results from OptGene (with biomass production as biological objective function) (Table 2). Using OptGene, only one single reaction deletion resulted in ethanol production: oxygen uptake rate (O2_UP). iStruF results showed several other potential reaction knock-out strategies besides anaerobic fermentation, including removing alternative fermentation routes towards lactate and acetate. Most iStruF results on single gene deletions are obvious and thereby demonstrate the feasibility of the method in this stage. Interestingly, the prediction for the wild-type phenotype seems more realistic when using iStruF, as it produces ethanol, whereas the wild-type using FBA does not.

**Table 2:** Predicted ethanol production for single reaction deletions in *S. cerevisiae* using OptGene in column two (Patil et al., 2005) and using iStruF in columns three and four.

Reaction Knockout	BO*DO	StruF^ETOHt^	Growth (%)
O2_UP	1.3	1.50	35
ALD4	0	0.56	100
GDH13	0	0.53	79
GDH2	0	0.48	86
KGD, LSC	0	0.47	97
IDH	0	0.42	99
ZWF1, SOL, GND	0	0.42	90
SDH	0	0.41	87
TKL2	0	0.41	88
RPE	0	0.41	89
MLS1	0	0.41	98
CIT13	0	0.40	96
PDA	0	0.39	96
PCK1, GCV1	0	0.38	99
GAD1, UGA1, UGA2	0	0.38	100
FBP1	0	0.37	99
ASP3	0	0.36	99
SHM12, wild-type	0	0.36	100

The biological objective (BO) is growth, the design objective (DO) ethanol production. Glucose uptake is 1. Growth is relative to the wild-type growth rate.

It is relevant to couple product formation to growth for a viable mutant. Depending on the mode of fermentation, a mutant may be chosen that exhibits fast or slow growth. If one aims to produce a compound in two-stage fermentation with a growth and a production phase, one may opt for a reversible knockout with low growth, because a high growth rate reduces the achievable product yield. If on the other hand, one aims to produce a compound while growing, a mutant with a high growth rate may be the better choice to obtain higher productivity. Amongst the viable mutants with enhanced ethanol production, growth was predicted to be between 79% and 100% of the wild-type rate. Deletion of one of the top ranked candidate reaction, GDH1, has been shown to experimentally contribute to a higher ethanol yield with good growth [40].

#### Succinate production in yeast

**Figure 6:**
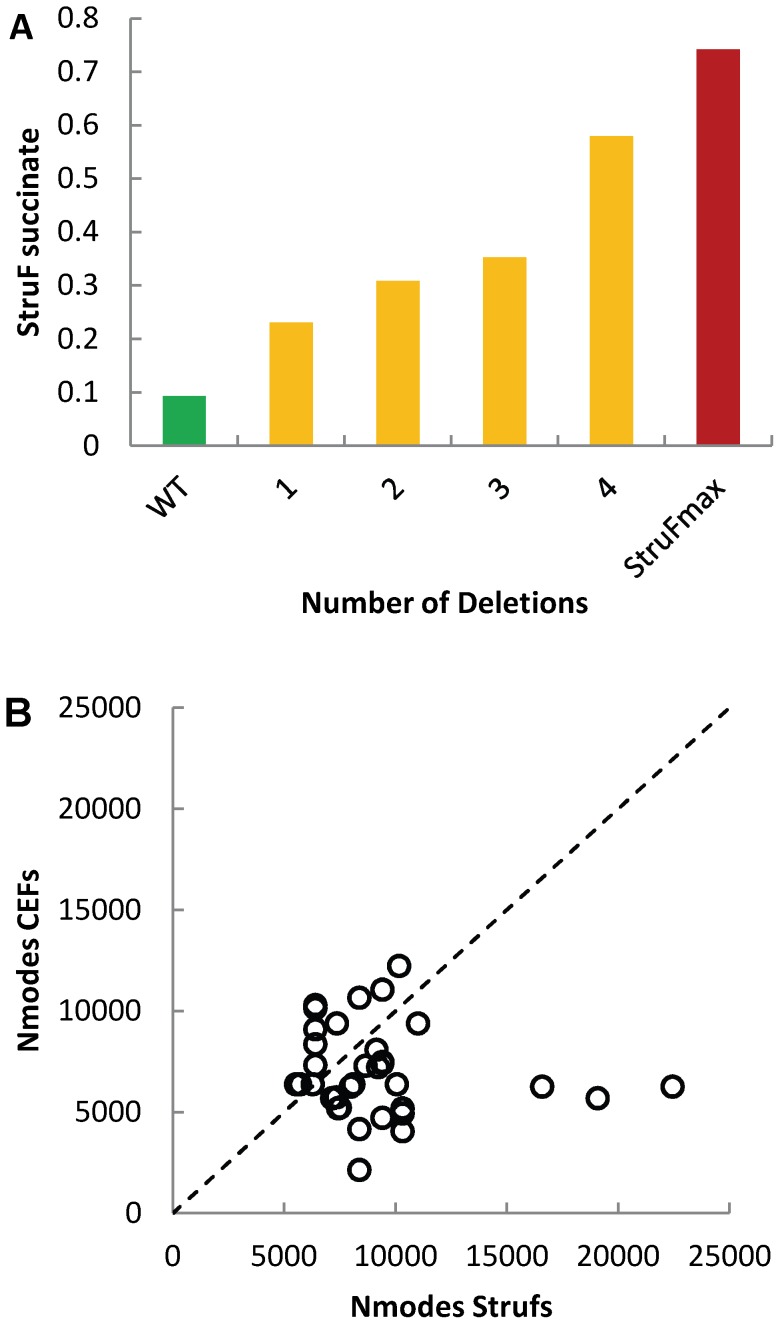
Results from iStruF for succinate production in yeast. **A.** Maximum structural flux for an increasing number of knockouts. **B.** Number of elementary modes of the top 50 ranked control effective fluxes versus number of elementary modes of the top 50 structural fluxes in triple knockouts.

The maximum theoretical structural flux towards succinate is 0.74 mole succinate/mole glucose while considering the biomass producing modes, whereas the structural flux for the wild-type is 0.093. Thus, theoretically, eight fold improvement in production (Fig. 6A) can be achieved at the expense of an 18% decrease in growth. In Fig. 6 and Supplement S4, we show the results for triple knockouts using structural fluxes compared with control effective fluxes. Amongst the top-ranked solutions predicted by structural fluxes, we found many solutions that enhance a flux through the glyxolate shunt (and reduce the flux through the TCA cycle) and solutions that reduce the formation of by-products like acetate. One of these knockouts has been validated *in vivo* as part of a deletion strategy to improve succinate production [41], others have been discussed as promising targets [4]. In particular, knockout of the reactions ALD6, SER, and SDH may be promising as it allows for a predicted increase in succinate production (three fold compared with wild-type) and a high growth (88% of wild-type growth). In fact, deletion of SER and SDH has already been verified in Otero [42].

## Conclusions

Systematic consideration of the contribution of each pathway towards the cellular biological objective leads to the concept of structural fluxes. We have shown here that these structural fluxes reflect *in vivo* flux measurements and predict preferred reaction directionalities on a given substrate. In future, we expect that structural fluxes can be further verified as more experimental flux measurements become available, spanning multiple gene knockout mutants, larger networks, and with higher accuracy. In addition, structural fluxes can be used for understanding the type of regulation occurring in a given reaction.

Building on the predictive power of structural fluxes, we present a formulation of a novel *in silico* metabolic engineering algorithm, iStruF, which is able to find solutions for metabolic engineering targets that couple growth with product formation while considering optimal as well as sub-optimal routes and their efficiency. These solutions were found to include targets that have been partially validated *in vivo*. Together, structural fluxes and iStruF constitute a novel and promising toolset for metabolic engineering.

## Supporting Information

Supplement S1 ***E. coli*** **model (ME Model).**
(PDF)

Supplement S2 **Alignment of fluxes.** Table S1, *E. coli*. Table S2, *S. cerevisiae*.
(PDF)

Supplement S3 **Biological objectives.**
(PDF)

Supplement S4 **Normalization and succinate production.** Table S3, CEFs vs. StruFs.
(PDF)

Supplement S5 **Structural fluxes based on generating vectors.** Table S4, Pearson correlations for StruFs, CEFs, and FBA in *E. coli*. Table S5, Pearson correlations for StruFs, CEFs, and FBA in *S. cerevisiae*. Fig. S1, Pearson correlations for StruFs in *E. coli* and *S. cerevisiae*. Fig. S2, Phenotypic phase plane analysis in *E. coli*. Fig. S3, Phenotypic phase plane analysis in *S. cerevisiae*. Fig. S4, Reaction participation in elementary modes vs. generating vectors.
(PDF)

Supplement S6 **True match rates yeast.** Table S6, True match rates yeast.
(PDF)

Supplement S7 **Thermodynamic feasibility.** Fig. S5, Thermodynamic constraints in central metabolism of *E. coli*. Table S7, Overview of classified reactions based on thermodynamics. References.
(PDF)
