# Supplementary material for: Identification of Metabolic Engineering Targets through Analysis of Optimal and Sub-Optimal Routes
Source: PLoS One. 2013 Apr 23;8(4):e61648. doi: 10.1371/journal.pone.0061648 (PMC3633962; doi:10.1371/journal.pone.0061648)
Supplement: Supplement S1 — E. coli model (ME Model). (PDF) [file pone.0061648.s001.pdf]

Supplement S1: *E. coli* model (ME Model)

The network model was reconstructed to represent *E. coli* growing on glucose minimal media. The starting point was the full model for the central carbon metabolism of *E. coli* [1]. This model was modified in the following way: biomass formation was modeled by acknowledging the metabolic drain from the central metabolic pathways [2]; and oxidative phosphorylation was lumped [3].

***E. coli* model reaction identifiers**	***E. coli* model reaction names**	**Reactions**
1	PTS	Glucose + PEP -> G6P + PYR
2	GLCt	Glucose[e] -> Glucose
3	HEX1	Glucose + ATP -> G6P + ADP
4	PGI	G6P <-> F6P
5	PFK	F6P + ATP -> F1,6P + ADP
6	FBP	F1,6P -> F6P
7	FBA	F1,6P <-> DHAP + G3P
8	TPI	DHAP <-> G3P
9	GAPDH	G3P + NAD <-> PGP + NADH
10	PGK	PGP + ADP <-> 3PG + ATP
11	PGM	3PG <-> 2PG
12	PGMT	G6P <-> G1P
13	ENO	2PG <-> PEP
14	PYK	PEP + ADP -> PYR + ATP
15	PPS	PYR + ATP -> PEP + AMP
16	G6PDH	G6P + NADP <-> 6PGL + NADPH
17	PGL	6PGL -> 6PGC
18	PGDH	6PGC + NADP -> Ru5P + NADPH + CO2
19	RPE	Ru5P <-> X5P
20	RPI	Ru5P <-> R5P
21	TK1	R5P + X5P <-> S7P + G3P
22	TA	S7P + G3P <-> E4P + F6P
23	TK2	X5P + E4P <-> F6P + G3P
24	PDH	PYR + CoA + NAD -> AcCoA + NADH + CO2
25	CS	AcCoA + OAA -> CIT + CoA
26	ACONT	CIT <-> ICT
27	ICDHy	ICT + NADP <-> 2-KG + NADPH + CO2
28	AKGD	2-KG + NAD + CoA -> SUCCoA + NADH + CO2
29	SUCCAS	SUCCoA + ADP <-> SUC + CoA + ATP

30	FRD	FUM + FADH -> SUC + FAD
31	SUCD	SUC + FAD -> FUM + FADH
32	FUM	FUM <-> MAL
33	MDH	MAL + NAD <-> OAA + NADH
34	CITL	CIT -> OAA + Acetate
35	EDD	6PGC -> 2KDPG
36	EDA	2KDPG -> PYR + G3P
37	PPC	PEP + CO2 -> OAA
38	ME1	MAL + NAD -> PYR + NADH + CO2
39	PPCK	OAA + ATP -> PEP + ADP + CO2
40	ICL	ICT -> Glyoxylate + SUC
41	MALS	Glyoxylate + AcCoA -> MAL + CoA
42	PTAr	AcCoA <-> ACP + CoA
43	ACKr	ACP + ADP <-> Acetate + ATP
44	ACS	Acetate + ATP + CoA -> AcCoA + AMP
45	LDH	PYR + NADH <-> Lactate + NAD
46	ADHE	AcCoA + 2 NADH <-> Ethanol + 2 NAD + CoA
47	PFL	PYR + CoA -> AcCoA + FOR
48	Biomass	0.968 PEP + 0.13 G6P + 3.648 PYR + 0.071 F6P + 0.107 G3P + 0.458 E4P + 0.528 R5P + 1.662 3PG + 2.531 AcCoA + 1.789 OAA + 1.616 2KG + 18.528 NADPH + 3.258 NAD + 42.869 ATP<br>-> Biomass + 2.531 CoA + 18.528 NADP + 3.258 NADH + 42.869 ADP + 2.844 CO2
49	v80	NADH + 0.5 O2 + 2 ADP -> NAD + 2 ATP
50	v81	FADH + 0.5 O2 + ADP -> FAD + ATP
51	ATPM	ATP -> ADP
52	v83	NADH + FAD -> NAD + FADH
53	ADK	AMP + ATP <-> 2 ADP
54	THD5	NAD + NADPH -> NADH + NADP
55	POX	PYR + FAD -> Acetate + FADH + CO2
56	ACt	Acetate -> Acetate[e]
57	CO2t	CO2 <-> CO2[e]
58	O2t	O2[e] <-> O2
59	FORt	FOR -> FOR[e]
60	ETOHt	Ethanol-> Ethanol[e]
61	SUCCt	SUC -> SUC[e]
62	LACt	Lactate -> Lactate[e]
63	PYRt	PYR -> PYR[e]
