# Supplementary material for: Identification of Metabolic Engineering Targets through Analysis of Optimal and Sub-Optimal Routes
Source: PLoS One. 2013 Apr 23;8(4):e61648. doi: 10.1371/journal.pone.0061648 (PMC3633962; doi:10.1371/journal.pone.0061648)
Supplement: Supplement S2 — Alignment of fluxes. Table S1, E. coli. Table S2, S. cerevisiae. (PDF) [file pone.0061648.s002.pdf]

Supplement S2: Alignment of fluxes

The metabolic models used for structural flux calculations and ^{13}C-MFA models are not identical. Because the yeast model discriminates between parallel reactions in different subcellular compartments while the ^{13}C-flux model does not, these reactions have been summed. Isozymes are summed taking into account their forward or backward directionality. Also, we used the same approach when there are two different enzymes carrying fluxes in opposite directions. Lumped reactions in the ^{13}C-MFA models were also compared with the separate reactions of the ME models. Tables S1 and S2 specify this alignment for *E. coli* and yeast.

In the alignment of the gene-reactions relations for the analysis of expression data, in the case of complexes we selected the single transcript with the lowest expression level.

Table S1. Alignment of the reactions for *E. coli*. Summation or subtraction of particular reactions is shown, corresponding to their most frequent directionalities across the given mutants.

**^{13}C-MFA Reaction [4]**	***E. coli* ME model reaction identifiers**	***E. coli* ME model reaction names**
Glucose + PEP -> G6P + PYR	2	GLCt
G6P <-> F6P	4	PGI
F6P -> F1,6P	5-6	PFK-FBP
F1,6P -> DHAP + G3P	7	FBA
DHAP -> G3P	8	TPI
G3P -> 3PG	9	GAPDH
3PG <-> PEP	11	PGM
PEP -> PYR	14-15	PYK-PPS
G6P -> 6PG	16	G6PDH
6PG -> Ru5P + CO2	18	PGDH
Ru5P -> X5P	19	RPE
Ru5P -> R5P	20	RPI
R5P + X5P <-> S7P + G3P	21	TK1
S7P + G3P <-> E4P + F6P	22	TA
X5P + E4P <-> F6P + G3P	23	TK2
PYR -> AcCoA + CO2	24	PDH
AcCoA + OAA -> CIT	25	CS
CIT -> ICT	26	ACONT
ICT -> 2-KG + CO2	27	ICDHy
2-KG -> SUC + CO2	28	AKGD
SUC -> FUM	31-30	SUCD1i-FRD
FUM -> MAL	32	FUM
MAL <-> OAA	33	MDH
PEP + CO2 <-> OAA	37-39	PPC-PPCK
MAL -> PYR + CO2	38	ME1
ICT -> Glyoxylate + SUC	40	ICL
Glyoxylate + AcCoA -> MAL	41	MALS
AcCoA -> Acetate	42-44	PTAr-ACS
CO2 -> (Evolution)	57	CO2t
6-PG -> G3P + PYR	36	EDA
PYR -> Lactate	45	LDH
AcCoA -> Ethanol	46	ADHE

Table S2. Alignment of the reactions for *S. cerevisiae*. Summation or subtraction of particular reactions is shown corresponding to their most frequent directionalities across the given mutants.

**^{13}C-MFA reaction identifier [5]**	**^{13}C-MFA Reaction [5]**	**Yeast ME model reaction identifiers [6]**	**Yeast ME model reaction names [6]**
1	GLC + ATP -> G6P	38	GLK
2	G6P -> P5P + 2 NADPH +CO2	25	ZWF
3	G6P -> F6P	24	PGI1
4	F6P + ATP -> 2T3P	23	FBA1
5	2P5P -> S7P + T3P	30	TKL
6	P5P + E4P -> F6P + T3P	32	TKL2
7	S7P + T3P -> E4P + F6P	31	TAL1
8	T3P -> Ser + NADH	72	SER333
9-10	Ser + NADH <-> Gly + C1	75	SHM12
12	T3P -> Pep + ATP + NADH	19	GPM
13	PEP -> cytPYR + ATP	17	PYK
14	mitPYR -> mitAcCoA + CO2 + NADH	14	PDA
15	mitOAA + mitAcCoA -> CIT	5+49	CIT13+CIT2
16	CIT -> OGA + CO2 + NADH	6	ACO
17	OGA -> SUC + CO2 + 0.5ATP + NADH	9	LSC
18	SUC -> FUM + NADH	10-42	SDH-OSM1
19	MAL -> mitOAA + NADH	12-50	MDH1-MDH2
20	FUM -> MAL	11	FUM1
21	MAL -> mitPYR + CO2 + NADPH	13	MAE
22	cytOAA + ATP -> PEP + CO2	16	PCK1
23	cytPYR + CO2 + ATP -> cytOAA	15	PYC
24	acetate + 2ATP -> cytAcCoA	3	ACS.
25	Acetaldehyde -> acetate + NADPH	2+43	ALD6+ALD4
26	Acetaldehyde + NADH -> ethanol	1-79	ADH1-AHD3
27	T3P + NADH -> glycerol	39	GPD
30	cytAcCoA -> mitAcCoA	4	CAT2
32	cytPYR -> Acetaldehyde + CO2	41	PDC
33	O2 + 2NADH -> 2P/O x ATP	33	NADHX
	Biomass	81	BIOMX05-AA-F03
