# Supplementary material for: Identification of Metabolic Engineering Targets through Analysis of Optimal and Sub-Optimal Routes
Source: PLoS One. 2013 Apr 23;8(4):e61648. doi: 10.1371/journal.pone.0061648 (PMC3633962; doi:10.1371/journal.pone.0061648)
Supplement: Supplement S3 — Biological objectives. (PDF) [file pone.0061648.s003.pdf]

Supplement S3: Biological Objectives

Famili et al. [7] found that growth is a good biological objective for *S. cerevisiae*, whereas Schuetz et al. [8] found that ATP generation is a good objective for *E. coli*. We independently investigated the validity of these biological objectives using structural fluxes with different degrees of importance of biomass and ATP in the cellular objective (given by α) and comparing these against the measured ^{13}C fluxes.

$$\boldsymbol{SF = \frac{1}{\alpha + 1} \cdot SF^{Biomass} + \alpha \cdot SF^{ATP}}$$

α=0 indicates that the objective is the cellular growth reaction; α =∞ that the objective is the ATP maintenance reaction. In accordance to Famili et al. [7] and Schuetz et al.[8], Figure 2A shows that ATP is a better biological objective for *E. coli* (using ^{13}C data from [4] as reference); conversely, biomass was found to be a better objective for *S. cerevisiae* in Fig. 2B in terms of Pearson correlation coefficients between the predicted structural fluxes and the measured 13C fluxes from [5]).
