# Supplementary material for: Identification of Metabolic Engineering Targets through Analysis of Optimal and Sub-Optimal Routes
Source: PLoS One. 2013 Apr 23;8(4):e61648. doi: 10.1371/journal.pone.0061648 (PMC3633962; doi:10.1371/journal.pone.0061648)
Supplement: Supplement S4 — Normalization and succinate production. Table S3, CEFs vs. StruFs. (PDF) [file pone.0061648.s004.pdf]

Supplement S4: Normalization and succinate production

CEF values tend to be larger for smaller networks. If the CEFs as such are used to find deletion targets for the production of a desired metabolite, the algorithm tends to minimize the network size, besides maximizing product formation. The algorithm thereby favors the deletion of reactions with a high reaction participation and this leads to a biased set of deletion targets as we show in a test case on triple reaction deletions for succinate production in yeast in Table S3. The use of CEFs as such leads to a different ranking of deletion targets, in which the top ranked solutions contain less pathways. Moreover, the predicted growth using CEFs was higher than wild-type growth in many cases, which seems unrealistic. These effects would be even more prominent for mutants with a higher number of knockouts. Concluding, as CEFs are not comparable across networks, normalization is necessary to obtain feasible flux predictions.

Table S3. Predicted succinate production for triple reaction deletions in *S. cerevisiae* using structural fluxes (StruFs) compared with control effective fluxes [9]. Glucose uptake is 1. Growth is relative to the wild-type growth rate. CEF values are normalized *a posteriori* for the sake of comparison. Nmodes is the number of remaining modes after deletion of the three reactions.

StruFs				CEFs			
Reaction Knockouts	$StruF^{succ}$	Growth %	Nmodes	Reaction Knockouts	CEF^{succ}	Growth %	Nmodes
ALD6,SDH,ZWF1	0.353	75.21	7492	SDH,TPI,ZWF1	1.2798	134.54	5237
ALD6,SDH,SOL	0.353	75.21	7492	SDH,TPI,SOL	1.2798	134.54	5237
ALD6,SDH,GND	0.353	75.21	7492	SDH,TPI,GND	1.2798	134.54	5237
ALD6,SDH,RPE	0.33498	74.872	7416	SDH,TPI,RPE	1.2531	134.78	5201
SDH,ZWF1,GLT1	0.32818	77.408	10308	SDH,TPI,TKL2	1.2192	135.05	5195
SDH,SOL,GLT1	0.32818	77.408	10308	SDH,TPI,GDH13	1.1375	135.71	4069
SDH,GND,GLT1	0.32818	77.408	10308	ADH1,SDH,MDH2	1.0487	130.18	4943
SDH,RPE,GLT1	0.31483	76.902	10154	SDH,TPI,GDH2	1.0101	121.73	12243
ALD6,SDH,TKL2	0.31184	73.491	7343	ADH1,SDH,MDH1	0.9647	126.44	5746
SDH,ZWF1,IDP2	0.30187	70.89	8368	ADH1,MDH1,MDH2	0.73877	126.79	4171
SDH,SOL,IDP2	0.30187	70.89	8368	ADH1,MDH1,ALD4	0.71739	120.92	2155
SDH,GND,IDP2	0.30187	70.89	8368	SDH,MDH2,ADH3	0.67099	137.62	10673
CAT2,SDH,ZWF1	0.30096	75.616	9402	SDH,MDH1,ADH3	0.65705	132.26	11069
CAT2,SDH,SOL	0.30096	75.616	9402	ADH1,MDH1,TPI	0.62902	112.17	4733
CAT2,SDH,GND	0.30096	75.616	9402	ALD6,SDH,ZWF1	0.50908	108.46	7492
SDH,ZWF1,CIT2	0.3002	75.436	9397	ALD6,SDH,SOL	0.50908	108.46	7492
SDH,SOL,CIT2	0.3002	75.436	9397	ALD6,SDH,GND	0.50908	108.46	7492
SDH,GND,CIT2	0.3002	75.436	9397	ALD6,SDH,RPE	0.47888	107.04	7416
SDH,ZWF1,SER333	0.29882	71.843	6390	SDH,ZWF1,GLT1	0.47057	111	10308
SDH,ZWF1,SER1	0.29882	71.843	6390	SDH,SOL,GLT1	0.47057	111	10308
SDH,ZWF1,SER2	0.29882	71.843	6390	SDH,GND,GLT1	0.47057	111	10308
SDH,SOL,SER333	0.29882	71.843	6390	MDH1,MDH2,ADH3	0.46722	135.67	9111
SDH,SOL,SER1	0.29882	71.843	6390	SDH,ZWF1,IDP2	0.45017	105.72	8368
SDH,SOL,SER2	0.29882	71.843	6390	SDH,SOL,IDP2	0.45017	105.72	8368
SDH,GND,SER333	0.29882	71.843	6390	SDH,GND,IDP2	0.45017	105.72	8368
SDH,GND,SER1	0.29882	71.843	6390	SDH,RPE,GLT1	0.44981	109.87	10154
SDH,GND,SER2	0.29882	71.843	6390	ALD6,SDH,TKL2	0.44369	104.56	7343
ADH1,SDH,ZWF1	0.29793	64.698	5702	SDH,ZWF1,SER333	0.44018	105.83	6390
ADH1,SDH,SOL	0.29793	64.698	5702	SDH,ZWF1,SER1	0.44018	105.83	6390
ADH1,SDH,GND	0.29793	64.698	5702	SDH,ZWF1,SER2	0.44018	105.83	6390
SDH,TKL2,GLT1	0.29729	75.282	10051	SDH,SOL,SER333	0.44018	105.83	6390
SDH,RPE,IDP2	0.29715	71.207	8100	SDH,SOL,SER1	0.44018	105.83	6390
SDH,RPE,SER333	0.29253	72.291	6273	SDH,SOL,SER2	0.44018	105.83	6390
SDH,RPE,SER1	0.29253	72.291	6273	SDH,GND,SER333	0.44018	105.83	6390
SDH,RPE,SER2	0.29253	72.291	6273	SDH,GND,SER1	0.44018	105.83	6390
ADH1,SDH,RPE	0.29174	64.771	5524	SDH,GND,SER2	0.44018	105.83	6390
SDH,RPE,CIT2	0.29036	75.181	9136	SDH,RPE,IDP2	0.43986	105.4	8100
SDH,MAE,ZWF1	0.29032	73.552	7358	CAT2,SDH,ZWF1	0.43155	108.43	9402
SDH,MAE,SOL	0.29032	73.552	7358	CAT2,SDH,SOL	0.43155	108.43	9402
SDH,MAE,GND	0.29032	73.552	7358	CAT2,SDH,GND	0.43155	108.43	9402
ALD6,SDH,SER333	0.29012	87.579	11003	SDH,ZWF1,CIT2	0.43046	108.17	9397
ALD6,SDH,SER1	0.29012	87.579	11003	SDH,SOL,CIT2	0.43046	108.17	9397
ALD6,SDH,SER2	0.29012	87.579	11003	SDH,GND,CIT2	0.43046	108.17	9397
CAT2,SDH,RPE	0.28893	75.148	9213	ADH1,MDH1,IDP2	0.42977	92.953	7236
ADH1,SDH,ADH3	0.28882	83.299	16580	SDH,RPE,SER333	0.42725	105.58	6273
ALD6,SDH,GDH2	0.2886	72.805	22437	SDH,RPE,SER1	0.42725	105.58	6273
SDH,TKL2,IDP2	0.28768	71.134	7985	SDH,RPE,SER2	0.42725	105.58	6273
SDH,MAE,RPE	0.28504	73.963	7144	ADH1,SDH,ZWF1	0.4272	92.77	5702
ALD6,SDH,GLT1	0.28462	101.52	19070	ADH1,SDH,SOL	0.4272	92.77	5702
Average	0.30238	74.5756	8644.33	Average	0.60344	113.324	7304
