# Supplementary material for: Identification of Metabolic Engineering Targets through Analysis of Optimal and Sub-Optimal Routes
Source: PLoS One. 2013 Apr 23;8(4):e61648. doi: 10.1371/journal.pone.0061648 (PMC3633962; doi:10.1371/journal.pone.0061648)
Supplement: Supplement S5 — Structural fluxes based on generating vectors. Table S4, Pearson correlations for StruFs, CEFs, and FBA in E. coli. Table S5, Pearson correlations for StruFs, CEFs, and FBA in S. cerevisiae. Fig. S1, Pearson correlations for StruFs in E. coli and S. cerevisiae. Fig. S2, Phenotypic phase plane analysis in E. coli. Fig. S3, Phenotypic phase plane analysis in S. cerevisiae. Fig. S4, Reaction participation in elementary modes vs. generating vectors. (PDF) [file pone.0061648.s005.pdf]

Supplement S5: Structural fluxes based on generating vectors

Introduction to generating vectors

GVs allow computation in polynomial time [10] and can be computed using the nullspace algorithm of METATOOL [11] or elmocomp [12] for larger metabolic networks. [13] show that CEFs computed from EMs and GVs are comparable, though if a reversible reaction is involved in forward and backward directions in the modes, its CEF computed from EMs tends to be underestimated compared to the CEF of the same reaction computed from GVs.

An issue that was encountered in the approach of using GVs is the need for re-computation of the minimal generating set after each gene deletion, because cancelation of reversible fluxes hides simple pathways [14]. To avoid this re-computation and to obtain a unique set of modes and a pointed flux cone, one can split all reversible reactions into two irreversible reactions, and compute the set of modes for such metabolic network. The result is again the set of generating vectors that coincides with the set of elementary modes. We propose a compromise in one step by restricting and splitting up reversible reactions until a pointed cone is obtained, so that re-computation after each reaction deletion is no longer necessary (main text, section 2.2.1). The proposed approach reduces the computational intensity compared to elementary modes and avoids re-computation of the minimal generating set, thus allowing the use of larger-scale networks compared to elementary modes, and it is a way to consider biologically relevant modes.

Procedure

The procedure for finding metabolic engineering targets consists of two main steps and is given in Fig. 1 using either EMs or GVs for larger-scale. Concerning the GVs, first, the structural fluxes are computed for the wild-type network as a measure that predicts the flux of all reactions across the network. Second, a search for mutants with an increased structural flux towards the target reaction is started.

In more detail, the procedure is as follows:

- Compute StruFs for the wild-type network including its forward and backward value for each reversible reaction (Eqs.1-3).
- Compute the reversibility score (Eq. 4) and restrict the reversiblities of a potentially reversible reaction if its reversibility score is smaller than 0.05; for the remaining reversible reactions: split up the reaction into a forward and backward reaction to obtain a modified metabolic network.
- Re-compute the generating vectors from the modified wild-type network. Note that, for the modified network, the minimal generating set is equal to the extreme rays. Compute the StruFs for this set of GVs.
- Search for mutants that have an increased structural flux for the target reaction by re-computing the structural fluxes for each knockout mutant.

Based on case studies on medium-scale networks, we chose 0.05 as a cut off for restricting the reactions reversibility. The cut off for the reversibility score represents a trade-off between prediction power and computational intensity. A lower cut off gives better predictions at the expense of increased computational effort. For the intended application to large-scale networks, fine-tuning of this cut off may be desired. As an example, upon deletion of the reactions ZWF or GND, the directionality of some of the reactions in the pentose phosphate pathway has to change to be able to supply the biomass building blocks and this should be reflected in the values of the StruFs in forward and backward directions.

Experimental validation

In this section, we justify the use of generating vectors instead of elementary modes in the computation of structural fluxes towards large-scale implementation by showing the prediction of the fluxes per mutant. Tables S4 and S5 show the correlation of StruFs with ^{13}C flux data as compared with the correlation of the same experimental data with fluxes estimated by FBA and CEFs, as well as StruFs calculated from EMs and GVs. It can be seen that structural fluxes are good predictors of intra-cellular fluxes in mutants for the central carbon metabolism of *E. coli* and *S. cerevisiae*. All p-values indicate a

significant correlation. Regarding the structural fluxes for *E. coli*, the Pearson correlation was at least 0.70. *S. cerevisiae* is a more complex micro-organism than *E. coli* and known to have a higher degree of regulation. Interestingly, the obtained correlations for *S. cerevisiae* were better than for *E. coli*: ≥ 0.82. FBA gave Pearson correlations of about 0.5 for yeast. Flux variability analysis under the constraint of optimal growth did not change the flux distribution of most fluxes (only of the futile cycles MDH1-MDH2, ADH1- ADH3, and OSM1-SDH) nor could improve the correlations. FBA gave Pearson correlations of about 0.3 for *E. coli*. These poor results are caused by a futile cycle through the glycolysis and pentose phosphate pathway. In flux variability analysis, these fluxes can have an infinite range of variation (constrained to -1000 and 1000 in our simulations). Slightly improved correlations can be obtained by setting additional constraints on the reversibility of for instance PGI. Compared to FBA, structural fluxes are the preferred choice. Compared to the original concept of CEFs, structural fluxes give a slightly better correlation. Note that, for comparison of fluxes across mutants (as demonstrated in the paper), the prediction power for structural fluxes was found to be far superior over control effective fluxes, because the latter are not comparable across networks.

To facilitate the use of large-scale networks, generating vectors were considered as an alternative for elementary modes. For the compromise, in which the directionality of the reactions was restricted or split up, the structural fluxes computed from the set of generating vectors or elementary modes are similar. Generating vectors are favorable for its lower computational time, though they tend to overestimate gene/reaction essentiality as the number of pathways is smaller .

Table S4. Correlations for predicted and measured fluxes per mutant in *E. coli* using ^{13}C flux measurements from [4].

Mutant	FBA		CEFs	88,109 EMs	StruFs	88,109 EMs	CEFs	9.272 GVs	StruFs	21,070 GVs
	p-value	Pearson r	p-value	Pearson r	p-value	Pearson r	p-value	Pearson r	p-value	Pearson r
WT	7,08E-05	0,652	1.33E-09	0.8509	4.53E-08	0.8221	9.00E-08	0.7953	4.49E-06	0.7399
HEX1	7,08E-05	0,652	5.02E-10	0.8613	1.73E-08	0.8354	9.14E-09	0.8279	2.42E-05	0.6995
PGI	4,17E-06	0,7239	8.34E-13	0.9129	1.88E-11	0.9102	-*	-	2.86E-09	0.8651
PPS	5,46E-06	0,7179	1.11E-10	0.8758	3.46E-09	0.8552	1.27E-08	0.8236	4.22E-07	0.7865
FBP	8,79E-06	0,7069	3.93E-09	0.8384	1.05E-07	0.8095	1.89E-07	0.7833	2.82E-05	0.6955
RPE	1,41E-04	0,631	2.25E-09	0.845	1.31E-08	0.8471	-	-	8.89E-06	0.7337
PGL	7,37E-13	0,908	5.41E-11	0.8756	7.01E-09	0.8468	1.44E-12	0.9035	7.28E-09	0.8463
G6PDH	5,63E-13	0,9153	3.59E-08	0.8092	1.08E-06	0.7784	3.88E-11	0.885	2.05E-06	0.7657
PGDH	3,06E-09	0,8489	1.02E-09	0.8611	4.80E-08	0.8297	1.42E-11	0.8994	1.49E-08	0.8455
TK1	1,06E-05	0,6939	4.02E-09	0.8307	6.90E-08	0.8159	-	-	1.18E-06	0.7675
TK2	1,36E-06	0,7473	1.07E-10	0.8762	2.89E-09	0.8572	-	-	1.17E-05	0.7177
PGMT	1,70E-05	0,6907	1.94E-09	0.8467	5.91E-08	0.8182	8.22E-08	0.7967	6.29E-06	0.7324
sum r		8,8878		10.2838		10.0257		6.7147		9.1953

*- indicates that the knockout was lethal and no growth was predicted in the simulations.

Table S5. Correlations for predicted and measured fluxes per mutant in *S. cerevisiae* using ^{13}C flux measurements from [5].

Mutant	FBA		CEFs	191,083 EMs	StruFs	191,083 EMs	CEFs	7,056 GVs	StruFs	64,370 GVs
	p-value	Pearson r	p-value	Pearson r	p-value	Pearson r	p-value	Pearson r	p-value	Pearson r
WT	7.64E-03	0.4852	3.48E-09	0.8551	1.18E-09	0.867	7.15E-09	0.8466	1.57E-10	0.8865
ADH3	7.32E-03	0.4874	1.40E-09	0.8652	1.24E-09	0.8665	6.76E-09	0.8473	2.52E-10	0.8822
ALD6	6.45E-03	0.4941	8.55E-08	0.8127	5.73E-08	0.8187	2.65E-07	0.7946	4.67E-09	0.8517
FUM1	-*	-	-	-	-	-	-	-	-	-
IDP1	7.64E-03	0.4852	2.47E-09	0.859	8.49E-10	0.8704	8.46E-09	0.8445	9.66E-11	0.8907
LSC1	8.91E-03	0.4769	4.18E-10	0.8774	1.07E-10	0.8898	1.71E-09	0.8631	4.44E-12	0.9139
MAE1	3.73E-03	0.5213	2.51E-09	0.8588	8.59E-10	0.8703	2.06E-09	0.861	3.43E-10	0.8793
MDH1	3.84E-03	0.5199	3.61E-10	0.8788	2.66E-10	0.8817	-	-	7.86E-13	0.9246
PCK1	7.97E-03	0.4829	1.90E-09	0.8619	5.59E-10	0.8746	3.41E-09	0.8553	5.14E-11	0.8959
PDA1	2.39E-04	0.6315	2.00E-11	0.9033	1.05E-11	0.908	2.00E-10	0.8843	7.33E-13	0.925
RPE1	6.99E-05	0.6701	4.01E-09	0.8535	2.79E-09	0.8576	-	-	6.09E-09	0.8485
SDH1	3.47E-01	0.181	3.69E-10	0.8786	8.70E-11	0.8916	6.21E-10	0.8736	7.78E-11	0.8925
ZWF1	7.95E-03	0.483	3.11E-09	0.8564	1.34E-09	0.8657	8.35E-09	0.8447	-	-
sum r		5.9185		10.3607		10.4619		8.515		9.7908

*- indicates that the knockout was lethal and no growth was predicted in the simulations.

As examples, Fig. S1A shows the predicted structural fluxes against the measured ^{13}C fluxes for *E. coli* and Fig. S1B for yeast on a log-log scale for ease of visualization. Each graph represents a particular mutant. The fluxes were normalized for the glucose uptake of each mutant. The dotted line represents the fit with its corresponding Pearson correlation r. Note that most fits do not cross the origin, as the structural fluxes represent a “capacity” for each flux, whereas the measurements reflect a particular situation, in this case batch fermentation (in the case of *E. coli* the data were obtained from a chemostat at 0.1 h^{-1}). The intercept can be interpreted as a “degree of regulation”. Using *E. coli* for instance, the measured acetate flux is zero at a low specific growth rate. The structural flux, on the other hand, is nonzero as it represents the capacity of the network. It can produce acetate, but not necessarily, reflected by a nonzero intercept. Also note that no structural fluxes were predicted for the FUM1 mutant in yeast, as no biomass producing modes were left upon its deletion. We expect that a larger-scale model (containing more transport reactions) would predict a viable FUM1 mutant.

Phenotypic phase plane analysis shows that the yields in the elementary modes and generating vectors have a similar distribution (Figs. S2-S3). As mentioned before, [13] showed that the CEFs computed from EMs and GVs are comparable, though if a reversible reaction is involved in forward and backward direction in the modes, CEF tends to be slightly different due to a different reaction participation in the modes. Figure S4 shows that the reaction participation is highly correlated for elementary modes and generating vectors with a Pearson correlation coefficient higher than 0.93. To compare each reaction participation of elementary modes with generating vectors, the split reactions (into forward and backward direction) were summed for the generating vectors.

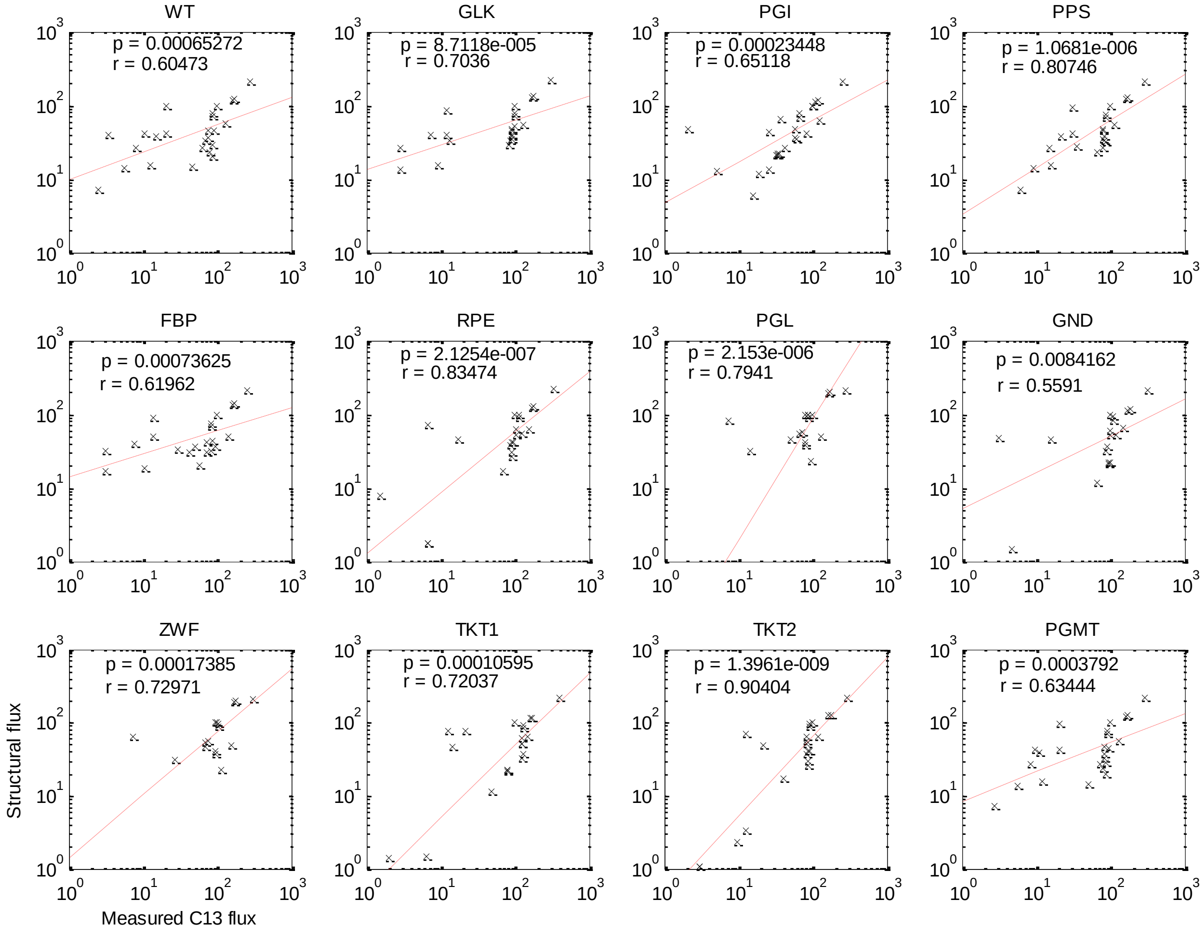

Fig. S1A. Pearson correlations for log transformed StruFs in *E. coli.* Predicted structural fluxes versus measured ^{13}C fluxes of *E. coli* for the wild-type and mutants with single gene deletions using EMs. 10^2 (100%) represents the glucose flux. The fluxes are shown on a log log-scale. Fluxes smaller than 1% of the glucose flux were removed from this plot. r is the Pearson correlation coefficient.

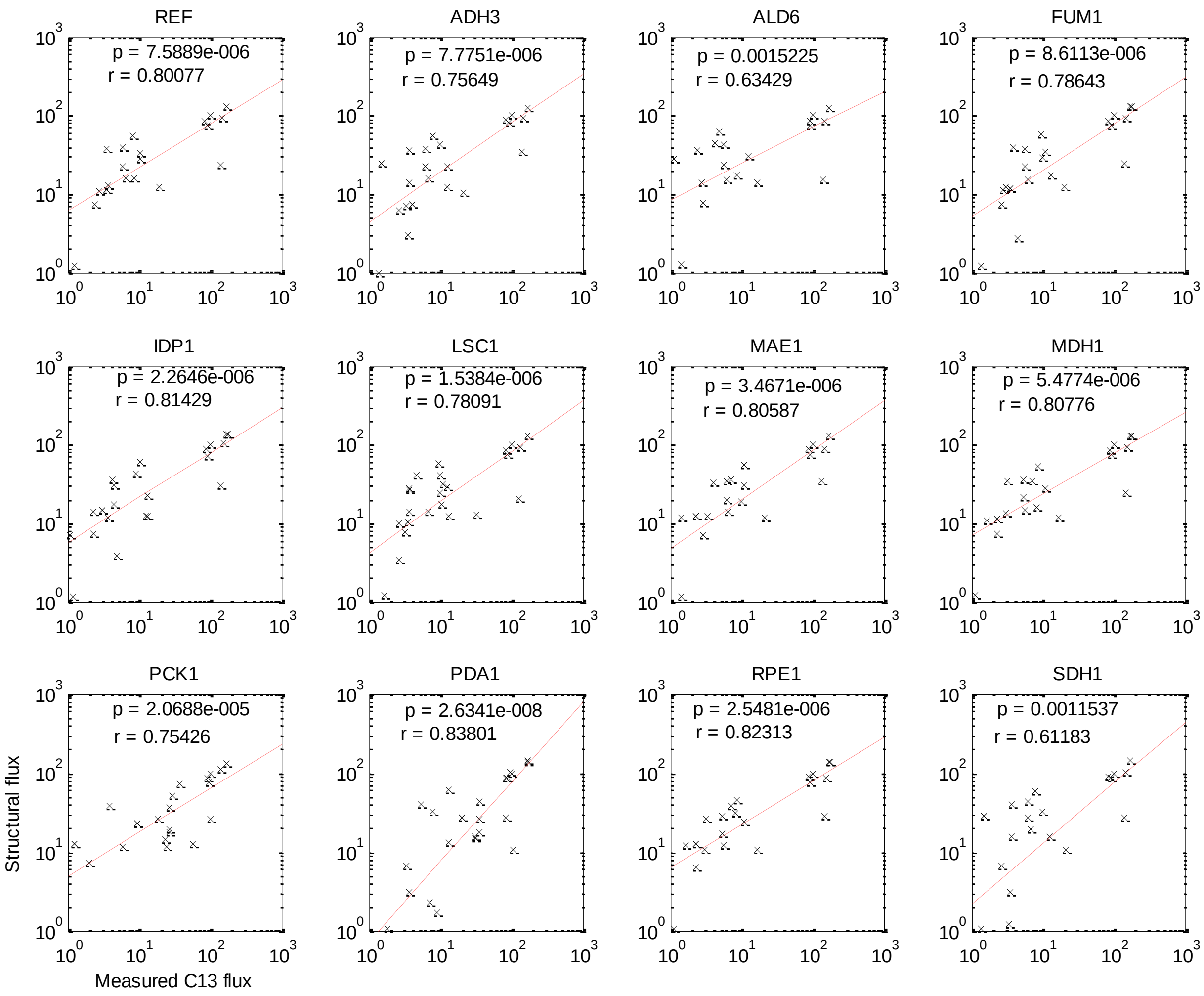

Fig. S1B. Pearson correlations for log transformed StruFs in *S. cerevisiae*. Predicted structural fluxes versus measured ^{13}C fluxes of *S. cerevisiae* for the wild-type and mutants with single gene deletions using EMs. 10^2 (100%) represents the glucose flux. The fluxes are shown on a log log-scale. Fluxes smaller than 1% of the glucose flux were removed from this plot. r is the Pearson correlation coefficient.

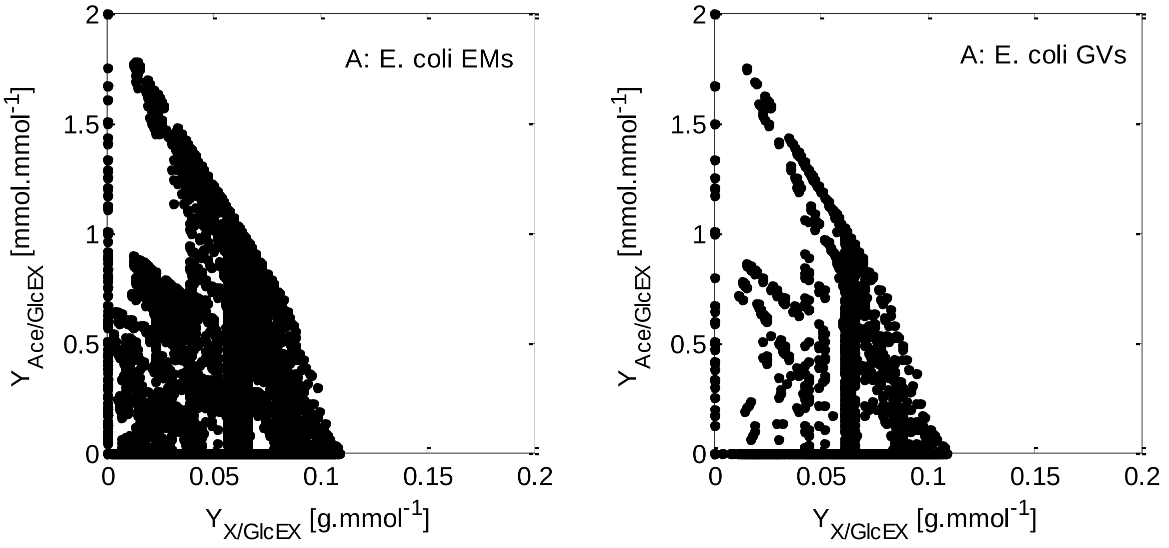

Fig. S2. Phenotypic phase plane analysis in *E. coli* . Biomass yield on glucose versus acetate yield on glucose for each pathway. **A.** Full set of elementary modes **B.** generating vectors.

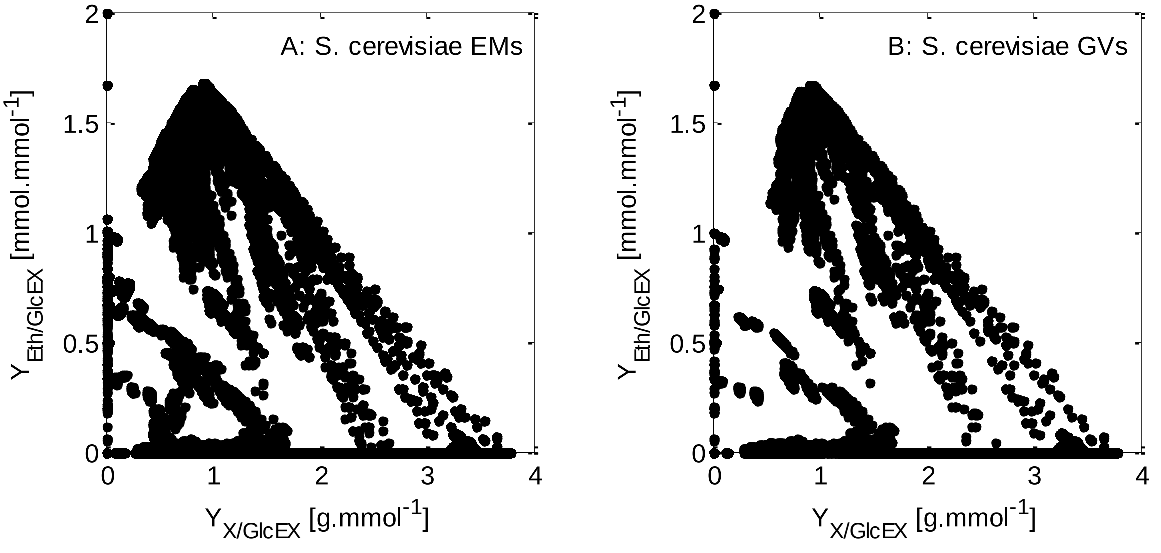

Fig. S3. Phenotypic phase plane analysis in *S. cerevisiae.* Biomass yield on glucose versus ethanol yield on glucose for each pathway. **A.** Full set of elementary modes. **B.** Generating vectors.

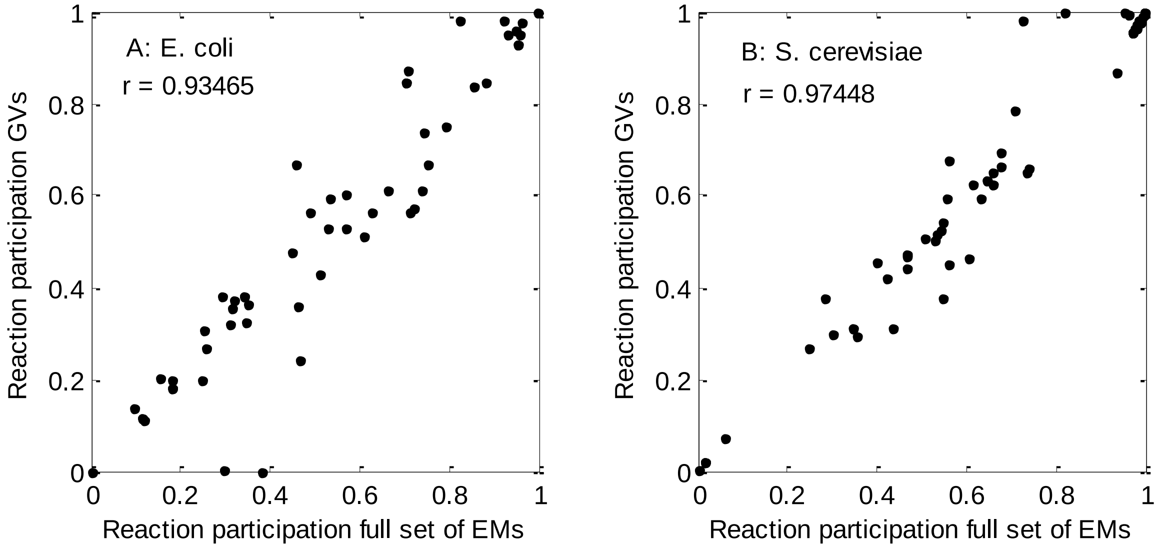

Fig. S4. Reaction participation in elementary modes vs. generating vectors. *r* represents the respective Pearson correlation coefficients. **A.** *E. coli.* **B.** *S. cerevisiae.*
