# Supplementary material for: Identification of Metabolic Engineering Targets through Analysis of Optimal and Sub-Optimal Routes
Source: PLoS One. 2013 Apr 23;8(4):e61648. doi: 10.1371/journal.pone.0061648 (PMC3633962; doi:10.1371/journal.pone.0061648)
Supplement: Supplement S6 — True match rates yeast. Table S6, True match rates yeast. (PDF) [file pone.0061648.s006.pdf]

Supplement S6: True match rates yeast

Table S6 shows the true match rates in *S. cerevisae*. Note the lower cut-off percentages for yeast in comparison to *E. coli* (Table 1) due to the lower impact on the fluxes of the deletions in yeast (for instance, there were no knockouts in yeast glycolysis whereas there where for *E. coli*).

Table S6. True match rate of the predicted flux changes (Eq. 6) and of the constant fluxes (Eq. 7) using a cut-off of 5% (and 25% in parenthesis) based on experimental data for *S. cerevisiae* using EMs, where 100% represents the glucose flux.

Reaction	True match rate TM^G [%]	Number of flux changes **greater** than cut-off	True match rate TM^S [%]	Number of flux changes **smaller** than cut-off
GLK	-*	0 (0)	85 (85)	78 (78)
ZWF	9 (-)	22 (0)	41 (65)	56 (78)
PGI1	15 (-)	26 (0)	52 (85)	52 (78)
FBA1	0 (-)	5 (0)	49 (78)	73 (78)
TKL	- (-)	0 (0)	58 (100)	78 (78)
TKL2	- (-)	0 (0)	58 (100)	78 (78)
TAL1	- (-)	0 (0)	58 (100)	78 (78)
SER333	- (-)	0 (0)	100 (100)	78 (78)
SHM12	- (-)	0 (0)	100 (100)	78 (78)
GPM	62 (-)	21 (0)	42 (85)	57 (78)
PYK	62 (-)	29 (0)	43 (85)	49 (78)
PDA	91 (92)	23 (12)	76 (100)	55 (66)
CIT13+CIT2	63 (91)	24 (11)	57 (99)	54 (67)
ACO	67 (82)	24 (11)	35 (84)	54 (67)
LSC	61 (90)	23 (10)	66 (100)	55 (68)
SDH-OSM1	70 (-)	23 (0)	38 (90)	55 (78)
MDH1-MDH2	71 (-)	21 (0)	30 (83)	57 (78)
FUM1	65 (-)	23 (0)	38 (90)	55 (78)
MAE	42 (-)	12 (0)	97 (100)	66 (78)
PCK1	- (-)	0 (0)	100 (100)	78 (78)
PYC	40 (-)	25 (0)	55 (99)	53 (78)
ACS.	100 (100)	12 (11)	45 (84)	66 (67)
ALD6+ALD4	78 (100)	18 (11)	48 (84)	60 (67)
ADH1-AHD3	60 (45)	53 (22)	44 (84)	25 (56)
GPD	9 (-)	22 (0)	59 (87)	56 (78)
CAT2	100 (100)	12 (12)	83 (100)	66 (66)
PDC	40 (17)	53 (12)	40 (82)	25 (66)
NADHX	59 (52)	39 (23)	79 (100)	39 (55)
BIOMX05-AA-F03	- (-)	0 (0)	85 (100)	78 (78)
Average	55 (77)	18 (5)	61 (92)	60 (73)

*The hyphen "-" indicates that no measurements were available.
