# Supplementary material for: Identification of Metabolic Engineering Targets through Analysis of Optimal and Sub-Optimal Routes
Source: PLoS One. 2013 Apr 23;8(4):e61648. doi: 10.1371/journal.pone.0061648 (PMC3633962; doi:10.1371/journal.pone.0061648)
Supplement: Supplement S7 — Thermodynamic feasibility. Fig. S5, Thermodynamic constraints in central metabolism of E. coli. Table S7, Overview of classified reactions based on thermodynamics. References. (PDF) [file pone.0061648.s007.pdf]

Supplement S7: Thermodynamic feasibility

We considered two aspects in our thermodynamic feasibility study. First, we checked for thermodynamic inconsistencies in the dataset or model and, second, we assigned reaction directionalities based on thermodynamic feasibilities in given environmental conditions. We evaluated the central carbon metabolism of *Escherichia coli* under two different conditions, growth on glucose and on acetate as the sole carbon sources.

Methods

The reaction directionalities in the model are imposed by the (ir)reversibilities of the enzymes. We aim to assign additional constraints based on thermodynamic grounds:

$$\Delta_r G_j < 0 \,\forall\, v_j > 0 \qquad \text{(S1)}$$

$$\Delta_r G_j > 0 \,\forall\, v_j < 0$$

When the estimated upper bound of the Gibbs energy of a particular reaction $\Delta_r G_j$ is negative, the net flux of reaction j can only proceed in the forward direction. Analogously, when the estimated lower bound of $\Delta_r G_j$ is positive, the reaction can only proceed in the reverse direction. We use the network-embedded thermodynamic (NET) analysis [15] together with the LINDO library to check for thermodynamic inconsistencies in the dataset and to assign reaction directionalities based on thermodynamic feasibilities in given experimental conditions. NET analysis determines the feasible range (i.e. upper and lower bounds) of free Gibbs energy of a particular reaction j using standard transformed Gibbs energies of formation $\Delta_f G_i^0$, metabolite concentrations c_i, and the respective stoichiometric coefficients $N_{i,j}$ (amongst others [16]):

$$\Delta_r G_j = \sum_i \mathrm{N}_{i,j} \Delta_f G_i^0 + RT ln\left(\prod_i c_i^{N_{i,j}}\right) \qquad \text{(S2)}$$

Intracellular metabolite concentrations are required to compute the actual Gibbs energies of reactions. These are widely unknown. Therefore, we employed broad physiological ranges for the unmeasured intracellular metabolite concentrations: 10^{-3} – 10 mM. For the measured metabolites, we employed their actual concentrations plus a 95% confidence interval if available.

Results

Net analysis

An overview of the classified reactions for each dataset is shown in Fig. S5 and Table S7. In general terms, it is important to emphasize that imposed constraints on reaction directionalities may be linked to actual measured concentrations, but may also be caused by bad measurements, may reflect faulty thermodynamic data or local differences in reactant activity, or may be induced by an incomplete or wrong model.

A reaction was considered to be feasible in the forward direction when the estimated upper bound of $\Delta_r G_j$ is negative and *vice versa*. Regarding glucose consumption, the directionalities for most reactions in the model were consistent with the corresponding $\Delta_r G_j$ values. The same was true for acetate. In four out of five datasets however, the TCA cycle seemed not fully feasible for our model due to the inability to generate NADH via malate dehydrogenase (MDH). This reaction, which is generally thought to synthesize oxaloacetate, was impossible given the necessary NAD/NADH ratio to allow the reaction to proceed. However, using the ubiquinone dependent malate quinone oxidoreductase (MQO), malate assimilation and the full TCA cycle were found to be feasible.

Several models representing the central metabolism of *E. coli* include MDH as the sole enzyme to catalyze the synthesis of oxaloacetate [1–3]. Thermodynamic analysis suggests the necessary extension of these models with the membrane-associated MQO that uses a ubiquinone, which is in accordance with [17]: “In view of the standard free energies of the MDH and MQO reactions, it would be likely that under physiological conditions MDH catalyzes the reduction of oxaloacetate and MQO oxidizes malate to oxaloacetate.” They showed that the reaction catalysed by MQO is largely used during exponential growth and that MQO and MDH are active at the same time in *E. coli*. On the other hand, they found that a mutant with deletion of the gene MQO had no growth defect. In addition, since the double mutant for MQO and MDH still grows on glucose, they suggested that an alternative route - other than the collective action of ME1, PPS, and PPC - has to exist to complete the TCA cycle.

Growth on glucose involves a net flux through glycolysis from DHAP to PEP. Growth on acetate on the other hand, involves the reverse (net flux through the gluconeogenesis). As the same enzymes are used in all cases, the thermodynamically favoured flux direction must change. Because reaction free energies are invariant physical properties, this can arise only from changes in the metabolite concentrations. As

mentioned in [18], *E. coli* grown in glucose has a substantially lower PEP/DHAP ratio than cells grown in acetate, favouring the glycolytic flux.

Figure S5 shows that, from a thermodynamic viewpoint, the uptake of glucose and thereby glycolysis is not feasible for growth on acetate only (red arrow). Both flux directions, however, seem feasible for growth on glucose as the sole carbon source. An issue of these particular datasets is that only three metabolites of glycolysis were measured for growth on glucose and two for growth on acetate, implying wide ranges for the other metabolites. Figure S5 also shows that the gluconeogenesis is infeasible for growth on glucose for the dataset of [19] (green arrows), which contains more relevant measurements (ENO is only feasible in the forward direction).

Thermodynamic analysis of the dataset of [20] imposed constraints on G6PDH and TK2 (black arrows). We compared the flux directions with C13 labelling experiments [21] and the constraints are consistent with the measured flux directionalities in glycolysis and the pentose phosphate pathway. Analysis of the dataset of [19] revealed constraints on ENO and ICDHy, which are consistent with the assumed fluxes in the glycolysis and TCA cycle.

However, analysis of the datasets of [19,20] imposed *tpi* to be feasible in backward direction only. This would imply flux from PEP to DHAP (gluconeogenesis), which is in disagreement with the assumed flux directionalities and with the thermodynamic results for ENO. In this case, we believe that the incorrectly implied reaction directionality is linked to inconsistencies in the measurements of the metabolites involved in this reaction (too high G3P/DHAP ratio).

Strikingly, in only one out of five datasets [4] the ATP synthase reaction, which is essential for optimal biomass growth, was classified as feasible in the forward direction (Table S7). ATP synthase activity may become unfavourable due to the failure of the proton gradient or due to an insufficient ADP/ATP ratio. More specifically, for the dataset of [18], the misclassification of this reaction may be due to an improper assumption of the unmeasured external pH at 7.0. However, we think that misclassification because of this is unlikely, since the three other datasets related to glucose-limited continuous cultivations revealed the same misclassification, although external pH was measured (7.0). A faulty assumption of the internal pH - it also has a decisive impact on the feasibility of ATP synthase - could be another cause of this misclassification, although it has been reported to be maintained at 7.6 for a

range of extracellular pH values [22]. The other cause, errors in the estimation of the ADP/ATP ratio, seems more likely, because the average ADP/ATP ratio in [4] (with feasible ATP synthase) was a factor 8 to 18 higher than in the other datasets.

Pathways and discussion

In the previous section, we found that the reactions catalysed by G6PDH, ICDHy, and ENO are thermodynamically feasible in the forward direction for growth on glucose as the only carbon source on specific datasets. We changed these reaction directionalities in the pathway analysis to compute only the feasible pathways. Restricting the flux directionalities of G6PDH and ICDHy did not exclude any pathways in the used network. Restricting ENO retained 68% of the original elementary modes. Further restricting TK2 yielded 43% feasible EMs.

As mentioned before, the imposed constraints on reaction directionalities can have many causes of uncertainties. There is a need for more and accurate measurement of (co)metabolite concentrations and standard Gibbs energies of the chemicals, because small changes in concentration can tip the thermodynamically favoured flux direction.

In addition, thermodynamics analysis gives constraints on the reaction reversibilities in particular conditions; it is questionable how these restrictions can be extrapolated to other conditions and mutants (in the view of our metabolic engineering strategy) without losing all assigned reaction directionalities. As an example, deletion of some reactions in the pentose phosphate pathway requires a flux in the reverse direction through TK2 in the mutant strains. Restriction of this reaction based on thermodynamic grounds from the wild-type, would give incorrect results for the mutants. Assignment of reaction directionalities based on reversibility scores, on the other hand, gives a measure for the potential variability of the fluxes in opposite directionalities. Consequently, a reversibility score above the cutoff would give rise to splitting up and thus keeping the reaction active in both directions in mutant strains. Overall, the largest number of restrictions in reaction reversibilities and the largest reduction in the number of pathways was obtained using the reversibility scores computed from structural fluxes, thus without the requirement for metabolite measurements, towards a biologically relevant description of phenotypes and metabolic engineering strategies.

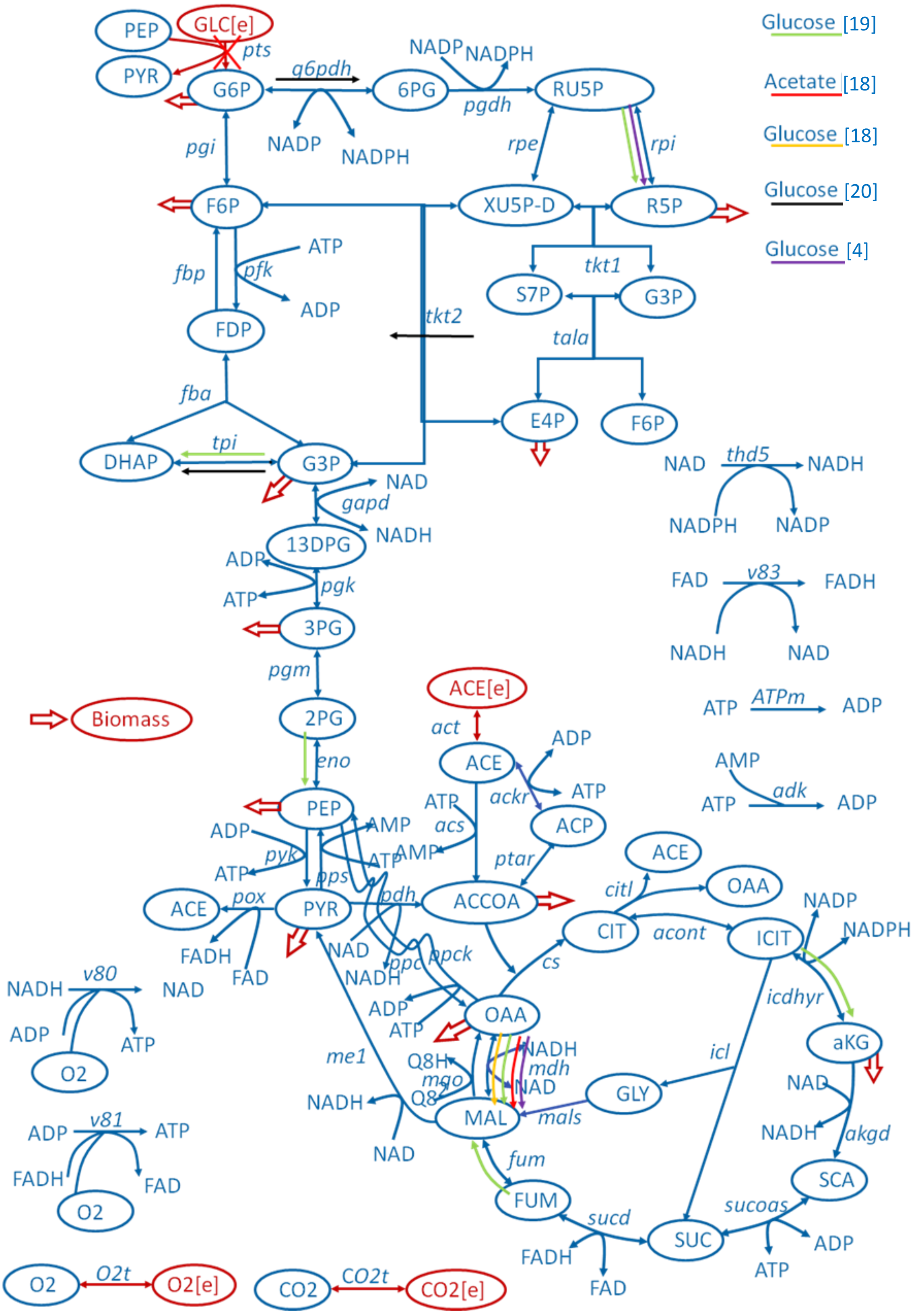

FIG. S5. Thermodynamic constraints in central metabolism of *E. coli*. The coloured arrows indicate the imposed thermodynamic reaction directionalities on specific datasets.

Table S7. Overview of classified reactions based on thermodynamics.

Dataset	Reaction	Bennet et al. [18]	Bennet et al. [18]	Ishii et al. [4]	Hoque et al. [19]	Chassagnole et al. [20]
Carbon source		Glucose	Acetate	Glucose	Glucose	Glucose
Feasibility	*pgm*: 3PG <==> 2PG				backward[4]	
	mdh: [c][1] MAL + NAD <==> H + NADH + OAA	backward	backward	backward	backward	[3]
	rpi: [c] RU5PD <==> R5P			forward	backward[4]	
	pts: GLC[e][2] + PEP[c] --> G6P[c] + PYR[c]		infeasible			
	tkt2: [c]E4P + XU5P-D <==> F6P + G3P					forward
	g6pdh: [c]G6P + NADP<==> 6PG + H + NADPH					forward
	tpi: [c] DHAP <==> G3P				backward[4]	backward[4]
	eno: [c]2PG <==> H_2O + PEP				forward	
	icdhyr: [c]ICIT + NADP + H_2O <==> aKG + CO_2 + NADPH				forward	
	ATPs4r: ADP[c] + (4) H[e] + PI[c] <==> ATP[c] + (3) H[c] + H_2O[c]	backward	backward		backward	backward

[1] [c] indicates the cytosolic compartment.

[2] [e] indicates the extracellular compartment.

[3] The blanks indicate no classification based on thermodynamics; feasible reaction.

[4] These reactions were misclassified due to faulty concentrations measurements. The blacklisted metabolites (2PG, RU5PD, and G3P in the dataset of [19]) should be removed before further analysis.

References

1. Suthers PF, Burgard AP, Dasika MS, Nowroozi F, Van Dien S, et al. (2007) Metabolic flux elucidation for large-scale models using C-13 labeled isotopes. Metab Eng 9: 387-405.

2. Fischer E, Zamboni N, Sauer U (2004) High-throughput metabolic flux analysis based on gas chromatography-mass spectrometry derived C-13 constraints. Anal Biochem 325: 308-316.

3. Carlson R, Srienc F (2004) Fundamental *Escherichia coli* biochemical pathways for biomass and energy production: Creation of overall flux states. Biotechnol Bioeng 86: 149-162.

4. Ishii N, Nakahigashi K, Baba T, Robert M, Soga T, et al. (2007) Multiple high-throughput analyses monitor the response of *E. coli* to perturbations. Science 316: 593-597.

5. Blank LM, Kuepfer L, Sauer U (2005) Large-scale C-13-flux analysis reveals mechanistic principles of metabolic network robustness to null mutations in yeast. Genome Biology 6.

6. Çakir T, Kirdar B, Onsan ZI, Ulgen KO, Nielsen J (2007) Effect of carbon source perturbations on transcriptional regulation of metabolic fluxes in *Saccharomyces cerevisiae*. BMC Syst Biol 1.

7. Famili I, Forster J, Nielson J, Palsson BO (2003) *Saccharomyces cerevisiae* phenotypes can be predicted by using constraint-based analysis of a genome-scale reconstructed metabolic network. Proc Natl Acad Sci 100: 13134-13139.

8. Schuetz R, Kuepfer L, Sauer U (2007) Systematic evaluation of objective functions for predicting intracellular fluxes in *Escherichia coli*. Mol Syst Biol 3.

9. Stelling J, Klamt S, Bettenbrock K, Schuster S, Gilles ED (2002) Metabolic network structure determines key aspects of functionality and regulation. Nature 420: 190-193.

10. Wagner C, Urbanczik R (2005) The geometry of the flux cone of a metabolic network. Biophys J 89: 3837-3845.

11. Pfeiffer T, Sanchez-Valdenebro I, Nuno JC, Montero F, Schuster S (1999) METATOOL: for studying metabolic networks. Bioinformatics 15: 251-257.

12. Jevremovic D, Trinh CT, Srienc F, Sosa CP, Boley D (2011) Parallization of Nullspace Algorithm for the computation of metabolic pathways. Parallel Computing 37: 261-278.

13. Larhlimi, A. (2008) New concepts and tools in constraint-based analysis of metabolic networks [dissertation]. Berlin: Freie Universitat Berlin.

14. Llaneras F, Pico J (2010) Which Metabolic Pathways Generate and Characterize the Flux Space? A Comparison among Elementary Modes, Extreme Pathways and Minimal Generators. J Biomed Biotechnol.

15. Zamboni N, Kummel A, Heinemann M (2008) anNET: a tool for network-embedded thermodynamic analysis of quantitative metabolome data. BMC Bioinformatics 9.

16. Stephanopoulos, G., Nielsen, and Aristidou, A. (1998) Metabolic engineering. San Diego: Academic Press.

17. van der Rest ME, Frank C, Molenaar D (2000) Functions of the membrane-associated and cytoplasmic malate dehydrogenases in the citric acid cycle of *Escherichia coli*. J Bacteriol 182: 6892-6899.

18. Bennett BD, Kimball EH, Osterhout, Rabinowitz JD (2009) Absolute metabolite concentrations and implied enzyme active site occupancy in *Escherichia coli*. Nature Chem Biol 5: 593-599. 10.1038/nchembio.186.

19. Hoque MA, Ushiyama H, Tomita M, Shimizu K (2005) Dynamic responses of the intracellular metabolite concentrations of the wild type and pykA mutant *Escherichia coli* against pulse addition of glucose or NH3 under those limiting continuous cultures. Biochem Eng J 26: 38-49.

20. Chassagnole C, Noisommit-Rizzi N, Schmid JW, Mauch K, Reuss M (2002) Dynamic modeling of the central carbon metabolism of *Escherichia coli*. Biotechnol Bioeng 79: 53-73.

21. Zhao J, Shimizu K (2003) Metabolic flux analysis of *Escherichia coli* K12 grown on C-13-labeled acetate and glucose using GG-MS and powerful flux calculation method. J Biotechnol 101: 101-117.

22. Shimamoto T, Inaba K, Thelen P, Ishikawa T, Goldberg EB, et al. (1994) The NhaB Na+/H+ antiporter Is essential for intracellular pH regulation under alkaline conditions in *Escherichia coli*. Journal of Biochemistry 116: 285-290.
